# Targeting circular RNA-Glra2 alleviates retinal neurodegeneration induced by ocular hypertension

**DOI:** 10.18632/aging.205108

**Published:** 2023-10-10

**Authors:** Ting Wang, Shuyan Li, Xiu-Miao Li, Chaopeng Li, Fang Wang, Qin Jiang

**Affiliations:** 1 Department of Ophthalmology, The Affiliated Huaian No. 1 Hospital of Nanjing Medical University, Huai’an, Jiangsu 223300, China; 2 Department of Ophthalmology and Optometry, The Affiliated Eye Hospital, Nanjing Medical University, Nanjing, Jiangsu 210000, China; 3 Department of Ophthalmology, Clinical Medical College of Shanghai 10th People’s Hospital of Nanjing Medical University, Nanjing, Shanghai 200072, China

**Keywords:** glaucoma, retinal neurodegeneration, circular RNA, retinal ganglion cell

## Abstract

Glaucoma is a leading cause of irreversible vision loss characterized by retinal neurodegeneration. Circular RNAs (circRNAs) have emerged as the potential biomarkers and therapeutic targets for neurodegenerative diseases. However, the expression profiling of circRNAs in glaucomatous neurodegeneration has not been fully understood. In this study, we built a glaucomatous neurodegeneration model via the injection of microbeads into anterior chamber. circRNA expression profile and bioinformatics analysis revealed that compared with normal retinas, 171 circRNAs were dysregulated in the glaucomatous retinas, including 101 up-regulated circRNAs and 70 down-regulated circRNAs. Detecting the level of circular RNA-glycine receptor α2 subunit gene (cGlra2) in aqueous humor made it possible to distinguish glaucoma patients from cataract patients. Silencing of cGlra2 protected against oxidative stress- or hydrostatic pressure-induced retinal ganglion cell (RGC) injury *in vitro*. Moreover, silencing of cGlra2 retarded ocular hypertension-induced retinal neurodegeneration *in vivo* as shown by increased TUJ1 staining, reduced reactive gliosis, decreased retinal cell apoptosis, enhanced visual acuity, and improved retinal function. cGlra2 acted as a miRNA sponge to regulate RGC function through cGlra2/miR-144/BCL2L11 signaling axis. Collectively, this study provides novel insights into the underlying mechanism of retinal neurodegeneration and highlights the potential of cGlra2 as a target for the diagnosis and treatment of glaucoma.

## INTRODUCTION

Retinal neurodegeneration is an important pathological process, which occurs in the pathogenesis of several ocular diseases, such as diabetic retinopathy and glaucoma [1]. Glaucoma is known as a neurodegenerative disease and a major cause of blindness globally. It is usually characterized by loss of retinal ganglion cells (RGCs) and axon degeneration [2]. Previous studies have revealed that increased intraocular pressure (IOP), neurotrophin deprivation, inflammation, and genetic/epigenetic changes are shown as the major risk factors of glaucoma [3, 4]. Currently, the major option for glaucoma treatment is lowering IOP. However, lowering IOP is not always effective for hindering visual loss in the patients with glaucoma. In addition, the surgery for glaucoma is not always successful due to wound fibrosis [5]. Thus, it is still required to further understand the underlying mechanism of retinal neurodegeneration and develop novel therapeutic strategies for glaucoma.

Circular RNAs (circRNAs) are characterized by the covalently closed-loop structures, which can regulate gene expression in several biological processes via sequestration of miRNAs/proteins, altering gene transcription, splicing, or translation [6]. circRNAs are abundantly and dynamically expressed in the nervous system and are shown as the potential regulators of nervous system development. Recent studies have also revealed the role of circRNAs in neurodegenerative diseases, such as Alzheimer’s disease, traumatic brain injury, and stroke [7, 8]. The retina and the brain share several common features, such as similar microvasculature, neuronal cell components, and gene regulatory network [9, 10]. We thus speculated that circRNAs also played critical roles in the process of retinal neurodegeneration. Previous studies have revealed that silencing of circZYG11B, circZNF609, or circZRANB1 plays a neuroprotective role in retinal neurodegeneration [11, 12]. A study has been conducted to identify glaucoma-related cicRNAs in a rat glaucomatous model [13]. In fact, circRNA information in rat genome is less clear than that in murine genome. Herein, we built a glaucomatous neurodegenerative murine model and determined circRNA expression profiles in this model.

In this study, circRNA expression profile and bioinformatics analysis were conducted to determine circRNA expression profiles in retinal neurodegenerative model induced by ocular hypertension. Following the identification of dysregulated circRNAs in the glaucomatous model, we focused on one circRNA, cGlra2, and investigated its role and mechanism in RGC injury and retinal neurodegeneration *in vitro* and *in vivo*. Moreover, we estimated the potential of cGlra2 for the diagnosis of glaucoma. Together, this study would improve our understanding of the pathogenesis of retinal neurodegeneration and highlight its diagnostic value and therapeutic potential in glaucoma.

## RESULTS

### Identification of differentially expressed circRNAs during retinal neurodegeneration induced by ocular hypertension

**Figure 1:**
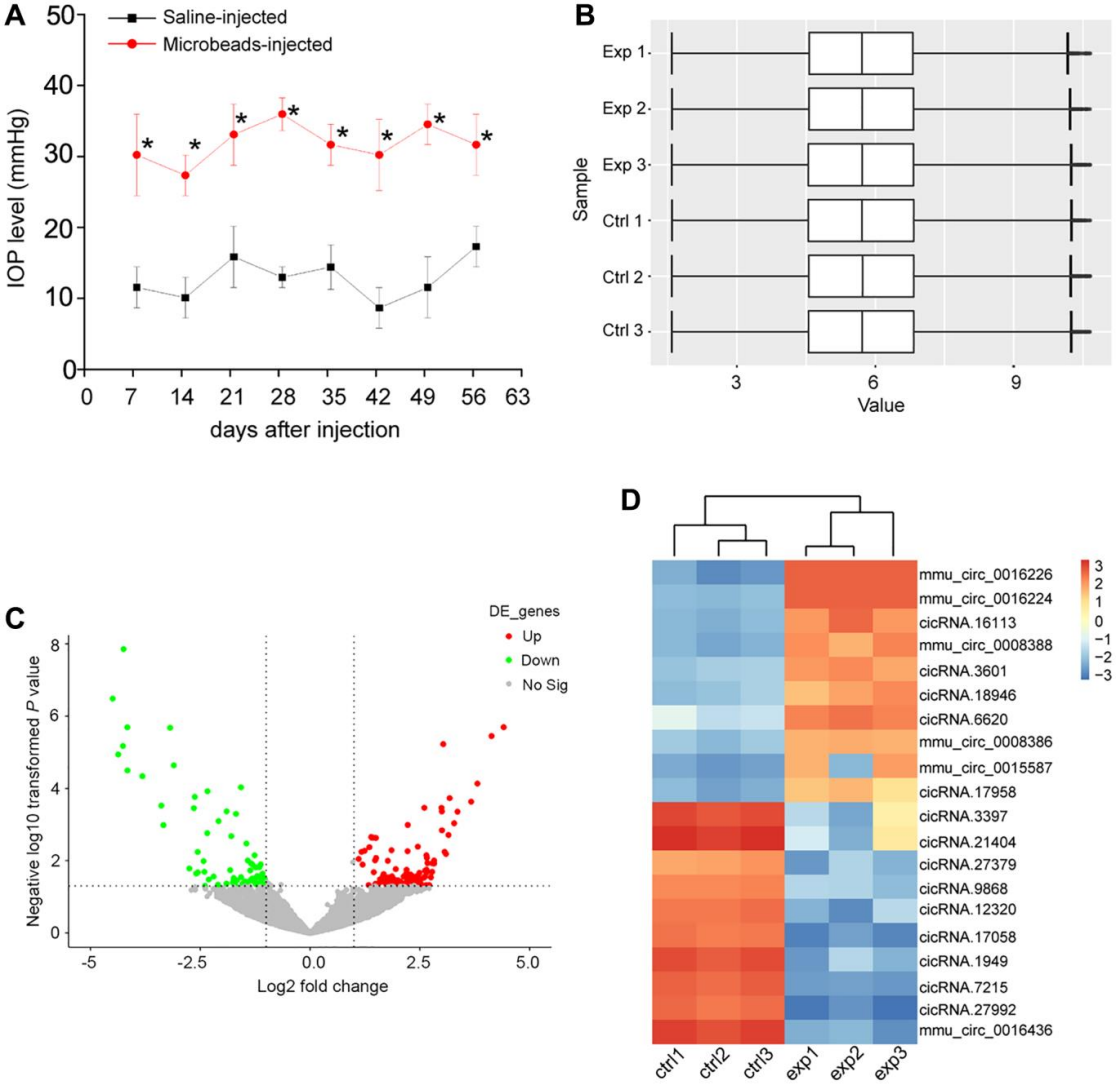
**Identification of differentially expressed circRNAs during retinal neurodegeneration induced by ocular hypertension.** (**A**) Retinal neurodegeneration was induced via anterior chamber injections of microbeads. The experimental group received anterior chamber injections of microbeads suspension to induce ocular hypertension, while the control group received anterior chamber injections of the same volume of saline suspension. Another injection of microbeads or saline solution was conducted 4-weeks after the initial injection. IOP levels in PBS buffer-injected retinas (Saline eyes) and microbeads-injected retinas (injected eyes) were shown (*n* = 5 animals; ^*^P < 0.05 vs. saline-injected retinas; Student *t* test). (**B**) Box plot was used to show the scale and expression distribution of circRNAs across different samples, which contained the boxes with a central line and two tails. The central line was data median and the tails were the upper and lower quartiles. (**C**) Volcano plots were plotted to show the differentially expressed circRNAs between microbeads-injected retinas and saline-injected retinas. (**D**) The heatmaps were generated via hierarchical cluster analysis to display the top 10 up-regulated and top 10 down-regulated circRNAs between microbeads-injected retinas and saline-injected retinas.

The mice received the anterior chamber injections of microbeads to induce ocular hypertension. In this model, microbeads-injection caused a marked enhancement in IOP levels (25–35 mmHg), which was about 3 times higher than the normal IOP levels. Then, a second microbeads-injection was conducted to maintain the increased IOP levels (Figure 1A). To investigate the change of circRNA expression profiling following ocular hypertension, we collected the microbeads-injected retinas and the saline-injected retinas at 8 weeks post-initial injection.

The box plot was used to assess whether the scale and distribution of microarray data across different samples were comparable. A box plot was plotted to display the distribution of the intensities of all samples after normalization. The result showed that the distribution of log2 ratios was nearly similar across different samples (Figure 1B), suggesting that the distribution of microarray data across different samples was comparable. Volcano plot was plotted to display the differential circRNA expression pattern between the microbeads-injected retinas and the saline-injected retina. 171 circRNAs were found to be dysregulated in microbeads-injected retinas (*P* < 0.05 and fold change >2). Among them, 101 circRNAs were up-regulated and 70 circRNAs were down-regulated (Figure 1C and Supplementary Table 1). Hierarchical clustering was conducted based on the differentially expressed circRNAs to hypothesize the relationships among different samples. The result revealed a distinguishable circRNA expression profiling across different samples. The microbeads-injected retinas were clustered into one branch, whereas the saline-injected retinas were clustered into the other branch (Figure 1D).

### Validation of detection accuracy of circRNA microarray by qRT-PCR assays

**Figure 2:**
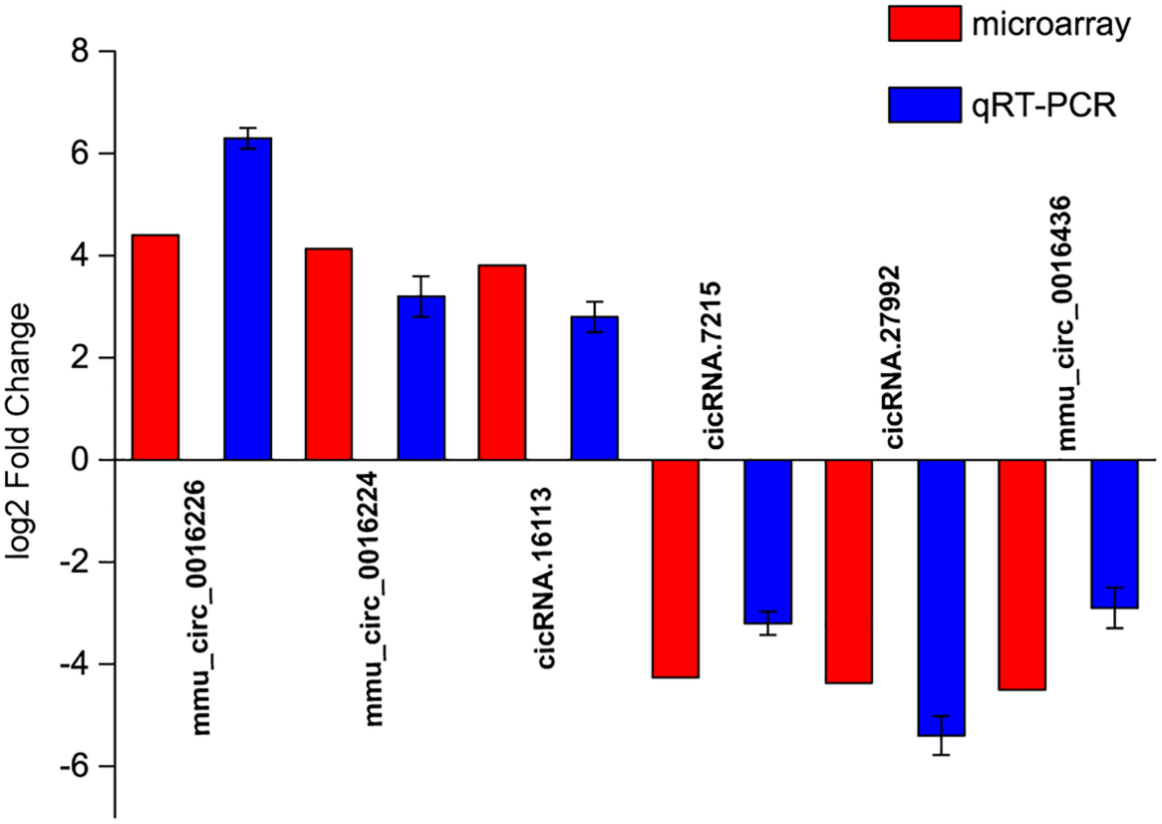
**Validation of detection accuracy of circRNA microarray by qRT-PCR assays.** qRT-PCR assays were conducted to compare the expression pattern of mmu_circ_0016226, mmu_circ_0016224, cicRNA.16113, cicRNA.7215, cicRNA.27992, and mmu_circ_0016436 between microbeads-injected retinas and saline-injected retinas. RNA samples were collected from microbeads-injected retinas (*n* = 12) and saline-injected retinas (*n* = 12) at 2-month after injection. RNA samples from 4 different retinas were pooled together as a biological replicate. Each group included 3 biological replicates.

To verify the results of circRNA microarrays, we selected 6 dysregulated circRNAs in the microbeads-injected retinas compared with the saline-injected retinas and detected their expression pattern by qRT-PCR assays. The results showed that the expression levels of mmu_circ_0016226, mmu_circ_0016224, and cicRNA.16113 were up-regulated in the microbeads-injected retinas, while the expression levels of cicRNA.7215, cicRNA.27992, and mmu_circ_0016436 were significantly down-regulated in the microbeads-injected retinas (Figure 2). The above-mentioned results indicate that there is a great expression consistency between circRNA microarrays and qRT-PCRs.

### Bioinformatics analysis of parental genes of dysregulated circRNAs by gene ontology (GO) analysis and Kyoto Encyclopedia of Genes and Genomes (KEGG) pathway analysis

**Figure 3:**
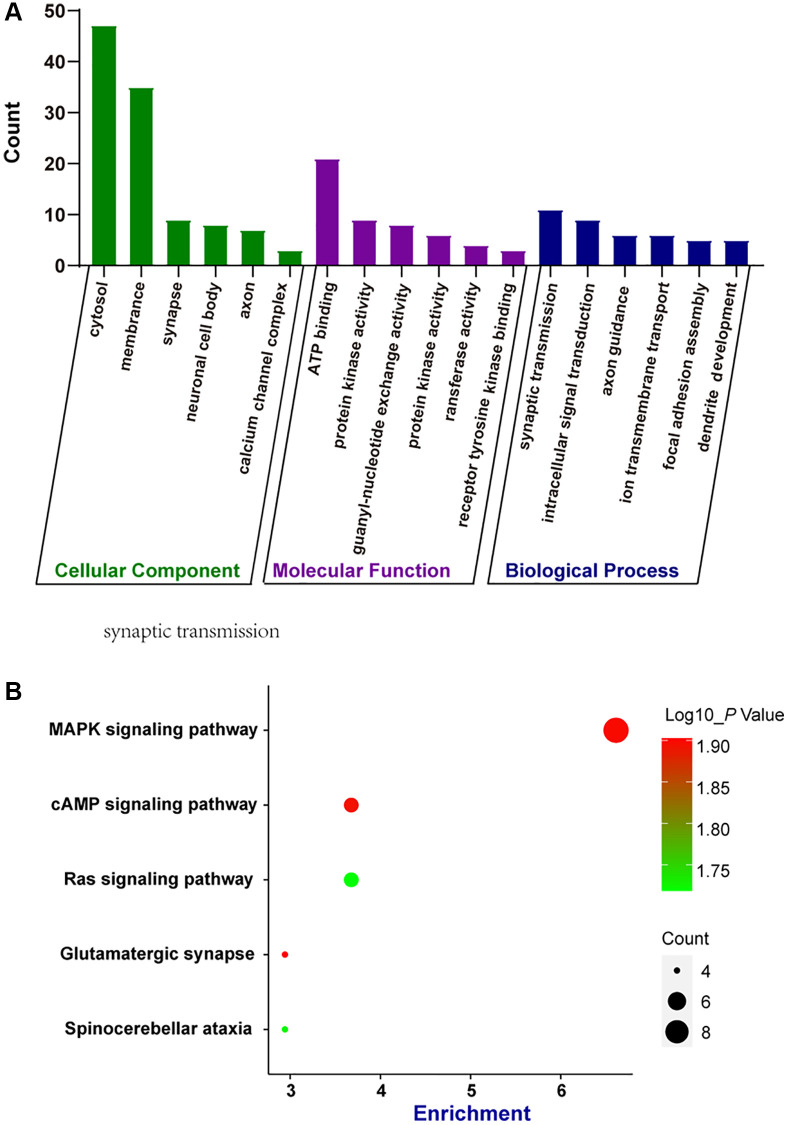
**Bioinformatics analysis of parental genes of circRNAs by GO enrichment analysis and KEGG pathway analysis.** (**A**) Gene ontology (GO) enrichment analysis included cellular components (CC), molecular functions (MF), and biological processes (BP). The functions of parental genes of the dysregulated circRNAs during retinal neurodegeneration were predicted by GO enrichment analysis. Fold enrichment analysis showed the regulation extent of the predicted functions compared with normal controls. (**B**) Bubble plot displayed the result of KEGG pathway analysis, showing the Top 5 signaling pathways predicted by parental genes of the dysregulated circRNAs during retinal neurodegeneration.

circRNAs are often produced from the parental genes via back-splicing. The functions of circRNAs may be predicted by the functions of their parental genes [14]. Here, we conducted GO enrichment analysis and KEGG pathway analysis of the parental genes of the dysregulated circRNAs during retinal neurodegeneration, which may provide great insights into the functions of circRNAs. GO enrichment analysis showed the enriched biological process (BP), cellular component (CC), and molecular function (MF) terms of the parental genes of dysregulated circRNAs. The top 5 enriched GO terms are shown in Figure 3A. The most enriched CC terms were related to cytosol. ATP binding was shown as the most enriched and meaningful MF term. As for BP, synaptic transmission was identified as the most enriched term. These results suggest that the parental genes of dysregulated circRNAs were tightly associated with cytosol, ATP binding, and synaptic transmission during retinal neurodegeneration. We further conducted KEGG analysis to identify the most enriched signaling pathways involved in dysregulated circRNAs-mediated network. The most enriched pathway was MAPK signaling pathway (Figure 3B), suggesting that the parental genes of dysregulated circRNAs during retinal neurodegeneration were enriched in MAPK signaling pathway.

### cGlra2 is shown as a potential biomarker for glaucoma

Since mmu_circ_0016226 is the most up-regulated circRNA during retinal neurodegeneration induced by ocular hypertension, we next determined whether mmu_circ_0016226 had a homologous gene in human genome. We identified a homologous gene of mmu_circ_0016226 in human genome, hsa_circ_0139862. Genome comparison analysis revealed that the sequence of mmu_circ_0016226 was strikingly similar to the sequence of hsa_circ_0139862 (Supplementary Figure 1). We named hsa_circ_0139862 and mmu_circ_0016226 as cGlra2 because they were produced from the parental gene of Glra2 mRNA. We then determined the expression distribution of cGlra2 in retinal tissues. FISH assays revealed that cGlra2 was mainly expressed in the ganglion cell layer (GCL) and inner nuclear layer (INL) (Supplementary Figure 2A). FISH assays were also conducted to compare the expression change of cGlra2 in ocular hypertension model (microbeads-injected retinas) and normal animals (saline-injected retinas). The results showed that the expression level of cGlra2 in ocular hypertension model was significantly higher than that in the normal animals (Supplementary Figure 2B). We also revealed that ocular hypertension induced by anterior chamber injections of microbeads led to the activation of MAPK signaling pathway as shown by increased levels of p-ERK, p-p38, and p-JNK, while intraocular injection of cGlra2 shRNA but not scrambled shRNA led to the inactivation of MAPK signaling pathway (Supplementary Figure 2C).

**Figure 4:**
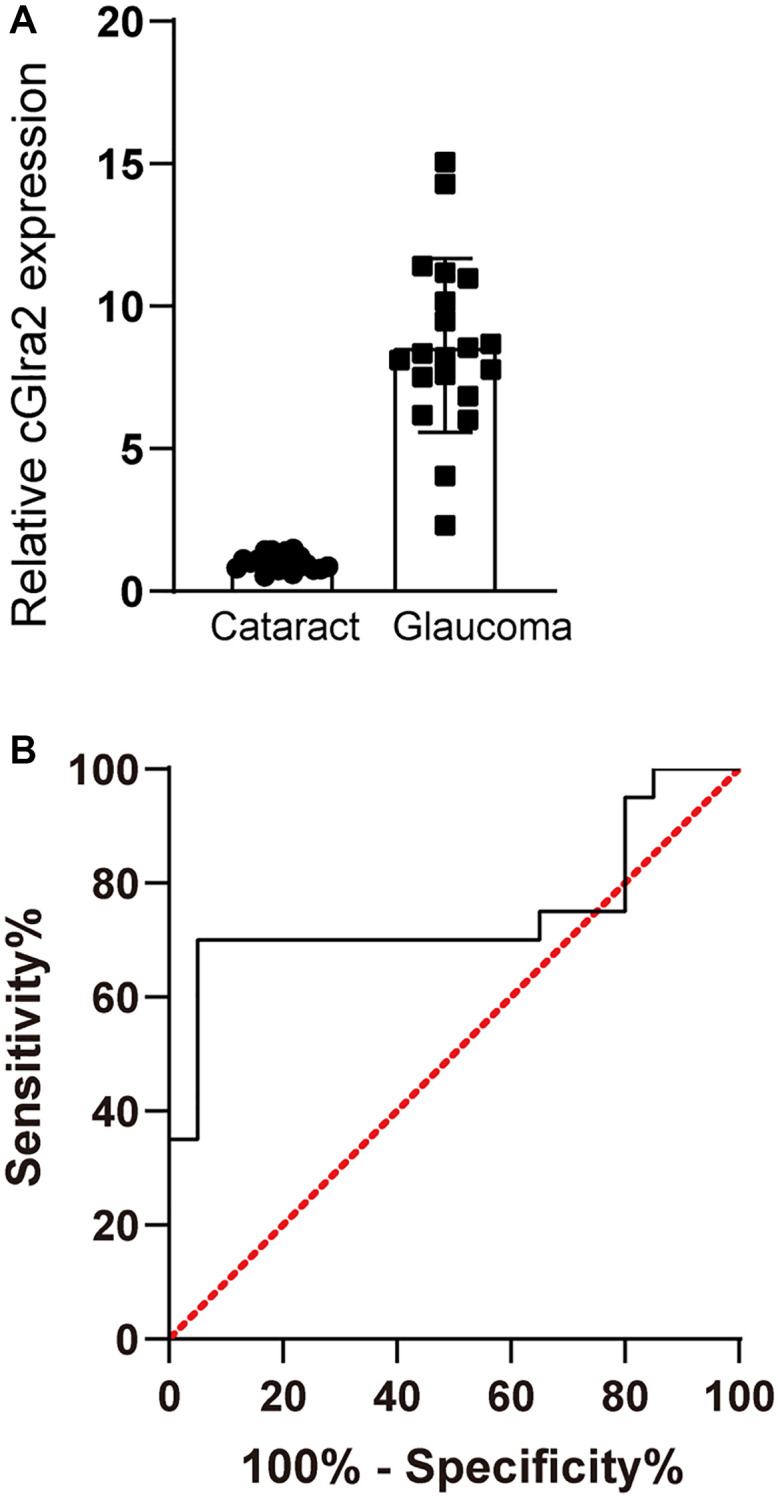
**cGlra2 is shown as a potential biomarker for glaucoma.** (**A**) AH samples were collected from glaucoma patients (*n* = 20) and cataract patients (*n* = 20). qPCR assays were conducted to detect the levels of cGlra2 expression. The significant difference was determined by Student’s *t*-test. (**B**) Receiver operating characteristic (ROC) analysis and AUC calculation was conducted to evaluate the diagnostic value of cGlra2.

Glaucoma is a retinal neurodegenerative disease characterized by optic nerve injury and RGC loss (Chang and Goldberg, 2012). We collected AH samples from the patients with glaucoma (*n* = 20) or the patients with cataracts (*n* = 20) undergoing surgery. The baseline demographics and characteristics of the involved patients were shown in Supplementary Table 2. cGlra2 levels in AH samples of glaucoma patients were significantly up-regulated compared with that in cataract patients (Figure 4A). ROC curves were generated for cGlra2. The specificity, sensitivity, and AUC (area under the curve) of cGlra2 for diagnosing glaucoma were 82.4%, 76.2%, and 0.75 (Figure 4B). Collectively, these results indicate that detection of cGlra2 level has a great potential for the diagnosis of glaucoma.

### Silencing of cGlra2 protects against oxidative stress-induced RGC injury

Increasing evidence has revealed that glaucoma is a complex multifactorial disease characterized by progressive RGC degeneration. RGC degeneration is the final common event in both human and experimental model of glaucoma [4]. We thus investigated whether cGlra2 played an important role in RGC injury *in vitro*. RGCs were primarily isolated and their purity was determined by immunofluorescence staining with TUJ1 and Thy 1.2. The results showed that most of the isolated cells were Thy 1.2-positive or TUJ1-positive, suggesting that most of the isolated cells are RGCs (Supplementary Figure 3).

**Figure 5:**
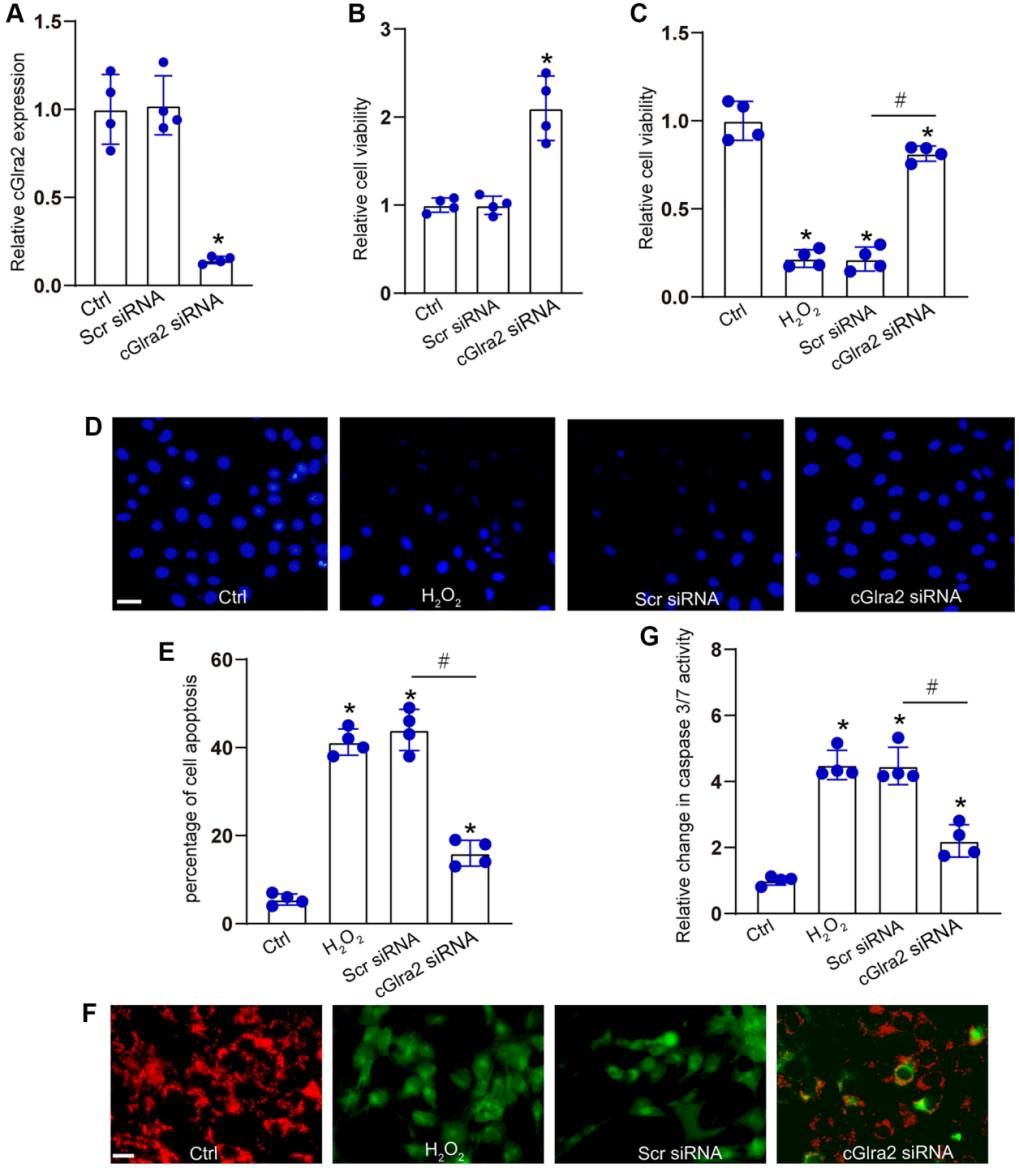
**Silencing of cGlra2 protects against oxidative stress-induced RGC injury.** (**A**) RGCs were transfected with scrambled (Scr) siRNA, cGlra2 siRNA, or left untreated (Ctrl) for 24 h. qRT-PCRs were conducted to examine the expression of cGlra2 (*n* = 4). (**B**) RGCs were transfected with scrambled (Scr) siRNA, cGlra2 siRNA, or left untreated (Ctrl) for 24 h. CCK-8 assays were performed to detect RGC viability (*n* = 4). (**C**–**G**) RGCs were transfected with Scr siRNA, cGlra2 siRNA, or left untreated (Ctrl) for 12 h and then exposed to H_2_O_2_ (100 μmol/L) to mimic oxidative stress for additional 36 h. CCK-8 assays were performed to detect RGC viability (**C**, *n* = 4). Hoechst staining and quantification analysis were performed to detect the changes of nuclei morphological characteristics of RGCs (**D** and **E**, *n* = 4, Scale bar: 50 μm). JC-1 staining was performed to detect the change of mitochondrial depolarization in RGCs (**F**, *n* = 4, Scale bar: 50 μm). Caspase 3/7 activity was performed to detect the degree of RGC apoptosis (**G**, *n* = 4). ^*^P < 0.05 vs. Ctrl group; ^#^*P* < 0.05 between the marked groups. All significance was examined using One-way ANOVA followed by Bonferroni’s post hoc test.

RGCs were transfected with cGlra2 siRNA to alter the levels of cGlra2 expression. qRT-PCR assays demonstrated that the levels of cGlra2 expression were markedly reduced following the transfection of cGlra2 siRNA in RGCs (Figure 5A). Under normal condition, CCK-8 assays demonstrated that transfection of cGlra2 siRNA led to a slight increase in the viability of RGCs (Figure 5B). Then, RGCs were exposed to H_2_O_2_ (100 μmol/L) to mimic the oxidative stress in the pathogenesis of glaucoma. CCK-8 assays showed that H_2_O_2_ treatment led to a marked reduction of RGC viability, while silencing of cGlra2 by the transfection of cGlra2 siRNA could alleviate oxidative stress-induced reduction of RGC viability (Figure 5C). Hoechst staining assays revealed that H_2_O_2_ treatment led to increased condensation and fragmentation of nuclei in RGCs, while silencing of cGlra2 significantly reduced the number of the condensate and fragmented nuclei in RGCs upon oxidative stress (Figure 5D, 5E). JC-1 staining showed that oxidative stress led to reduced mitochondrial depolarization in RGCs as shown by increased green signaling, while silencing of cGlra2 could reverse oxidative stress-induced reduction of mitochondrial depolarization as shown by increased red signaling (Figure 5F). Caspase 3/7 activity assay showed that silencing of cGlra2 could reverse oxidative stress-induced enhancement of caspase 3/7 activity (Figure 5G). Collectively, silencing of cGlra2 can protect against oxidative stress-induced RGC injury *in vitro*.

### Silencing of cGlra2 prevents against hydrostatic pressure-induced RGC injury *in vitro*

**Figure 6:**
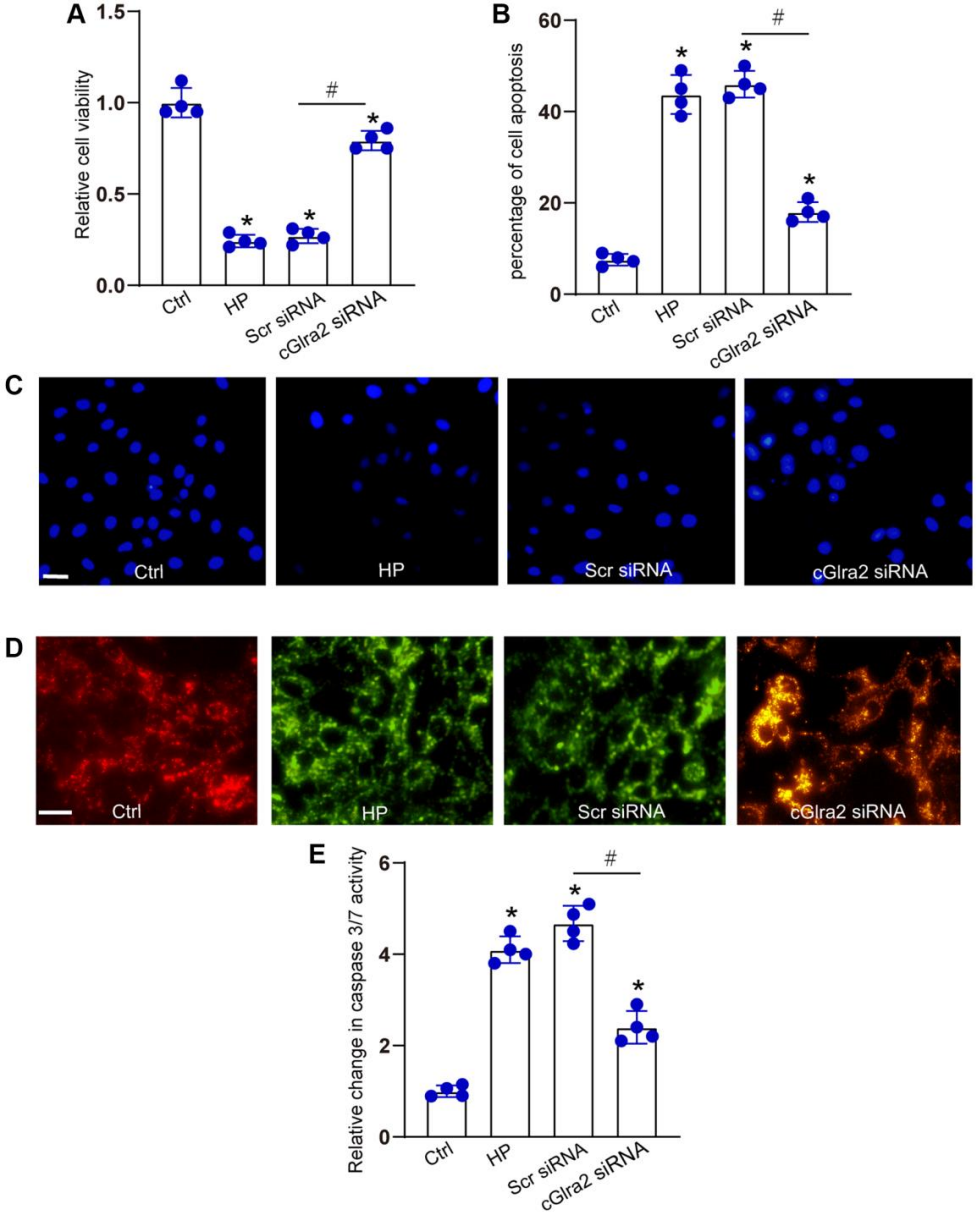
**Silencing of cGlra2 protects against hydrostatic stress-induced RGC injury.** RGCs were maintained under the elevated hydrostatic pressure (70 mm Hg) to induce hydrostatic stress. RGCs maintained at the ambient pressure were taken as the control group. RGCs were transfected with Scr siRNA, cGlra2 siRNA, or left untreated (Ctrl) for 12 h, and then accepted hydrostatic stress for additional 36 h. CCK-8 assays were performed to detect the viability of RGCs (**A**, *n* = 4). Hoechst staining and quantification analysis was performed to detect the changes of nuclei morphological characteristics of RGCs (**B** and **C**, *n* = 4, Scale bar: 50 μm). JC-1 staining was performed to change of mitochondrial depolarization in RGCs (**D**, *n* = 4, Scale bar: 50 μm). Caspase 3/7 activity was performed to detect the degree of RGC apoptosis (**E**, *n* = 4). ^*^P < 0.05 vs. Ctrl group; ^#^*P* < 0.05 between the marked groups. All significance was examined using the One-way ANOVA followed by Bonferroni’s post hoc test.

IOP is regulated by the production and outflow of aqueous humor. Excessive aqueous humor in glaucoma can lead to elevated hydrostatic pressure and induce RGC apoptosis [28, 37]. We then determined the effects of cGlra2 silencing on hydrostatic pressure-induced RGC injury *in vitro*. RGCs were maintained under the elevated hydrostatic pressure (70 mm Hg) to induce hydrostatic stress. RGCs maintained at ambient pressure were taken as the control group. CCK-8 assays demonstrated that transfection of cGlra2 siRNA alleviated hydrostatic stress-induced reduction of RGC viability (Figure 6A). Hoechst staining assays showed that hydrostatic stress significantly increased the number of the condensate and fragmented nuclei in RGCs, while silencing of cGlra2 could reduce the number of condensate and fragmented nuclei induced by hydrostatic stress (Figure 6B, 6C). JC-1 staining showed that hydrostatic stress led to decreased mitochondrial depolarization in RGCs, but silencing of cGlra2 reversed hydrostatic stress-induced reduction of mitochondrial depolarization (Figure 6D). Caspase 3/7 activity assays revealed that silencing of cGlra2 reduced hydrostatic stress-induced enhancement of caspase 3/7 activity (Figure 6E). Collectively, silencing of cGlra2 can protect RGCs against hydrostatic stress-induced injury *in vitro*.

### Silencing of cGlra2 alleviates ocular hypertension-induced retinal neurodegeneration *in vivo*

**Figure 7:**
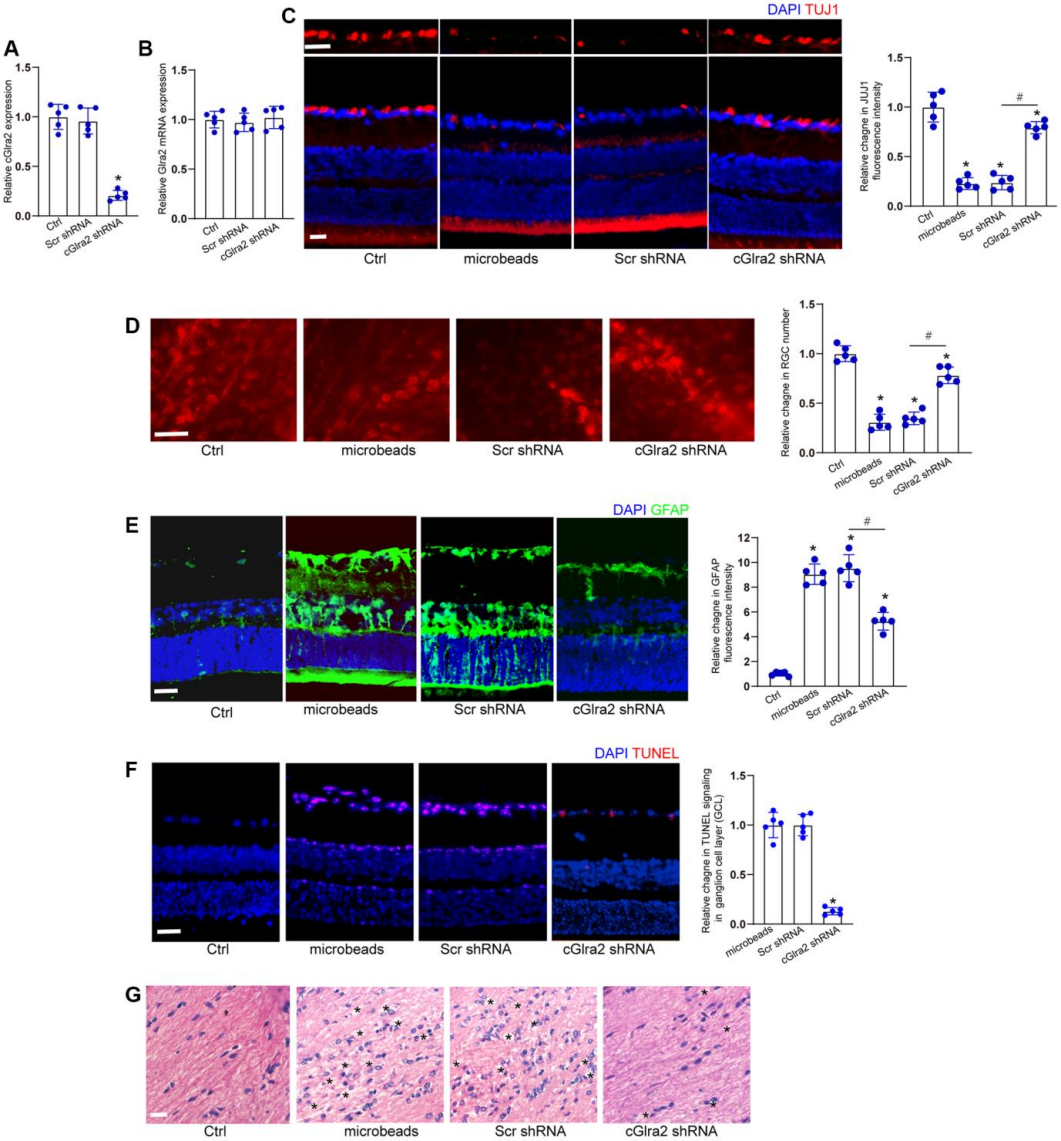
**Silencing of cGlra2 alleviates retinal neurodegeneration *in vivo***. (**A**, **B**) C57BL/6 mice received intravitreous injections of cGlra2 shRNA, scrambled (Scr) shRNA, or left untreated for 14 days. qRT-PCRs were performed to examine the expression levels of cGlra2 (**A**, *n* = 5 animals) and Glra2 mRNA (**B**, *n* = 5 animals). (**C**) Normal retinas (Ctrl), microbeads-injected retinas, microbeads-injected retinas plus Scr shRNA injection, or microbeads-injected retinas plus cGlra2 shRNA injection were stained with TUJ1 to label RGCs. Scale bar, 20 μm; *n* = 5 animals. (**D**) Retinal whole-mounts following TUJ1 staining were observed from peripheral area. RGC survival was calculated by dividing the average number of TUJ1-positive cells in one field in the injured retina by that in control (Ctrl) retina (*n* = 5 animals, Scale bar: 20 μm). (**E**) Immunofluorescence staining with GFAP was conducted to detect retinal neurodegeneration at 2-month following microbeads injection (*n* = 5 animals, Scale bar: 20 μm). Green: GFAP-positive cells; Blue: DAPI. (**F**) TUNEL assays were performed to detect retinal apoptosis at 2-month following anterior chamber injections of microbeads (*n* = 5 animals, Scale bar: 50 μm). Red: TUNEL-positive cells; Blue: DAIP. (**G**) Degeneration of RGC axons was detected by H&E staining (*n* = 5 animals, Scale bar: 20 μm). Three photographs were taken at 40 × magnification for each nerve (photograph from the proximal, central, and distal portion of optic nerve). “^*^” in Figure 7G indicated the swellings in RGC axons. ^*^P < 0.05 vs. Ctrl group; ^#^*P* < 0.05 between the marked groups; One-way ANOVA followed by the post hoc Bonferroni test.

We investigated whether the intervention of cGlra2 expression could alleviate ocular hypertension-induced retinal neurodegeneration *in vivo*. qRT-PCR assays revealed that intravitreous injection of cGlra2 shRNA led to a marked reduction of cGlra2 expression in the retinas, but had no effects on the expression of Glra2 mRNA (Figure 7A, 7B). FISH assays revealed that injection of microbeads led to increased Glra2 expression, while Glra2 knockdown led to reduced Glra2 expression in the retinas especially in GCL layer and INL layer (Supplementary Figure 4).

Immunofluorescence staining revealed that compared with scrambled shRNA-injected retinas, injection of cGlra2 shRNA reduced the degree of ocular hypertension-induced RGC injury as shown by increased TUJ1 staining (Figure 7C). We further conducted retinal whole-mount immunofluorescence to detect the survival status of RGCs. The results also demonstrated that injection of cGlra2 shRNA contributed to RGC survival as shown by increased RGC number compared with scrambled shRNA-injected retinas (Figure 7D).

Meanwhile, the retinas were stained with GFAP antibody to detect the degree of reactive gliosis. cGlra2 silencing led to reduced retinal reactive gliosis as shown by reduced GFAP staining (Figure 7E). TUNEL assays showed that cGlra2 silencing protected against ocular hypertension-induced retinal cell injury especially in GCL layer as shown by decreased TUNEL staining (Figure 7F). Degeneration of RGC axons was also detected following ocular hypertension. Hematoxylin-eosin (HE) staining revealed that silencing of cGlra2 alleviated ocular hypertension-induced injury of RGC axons as shown by less swellings in RGC axons compared with scrambled shRNA-injected retinas (Figure 7G). Collectively, silencing of cGlra2 alleviates ocular hypertension-induced retinal neurodegeneration *in vivo*.

### Silencing of cGlra2 alleviates visual acuity and retinal function

**Figure 8:**
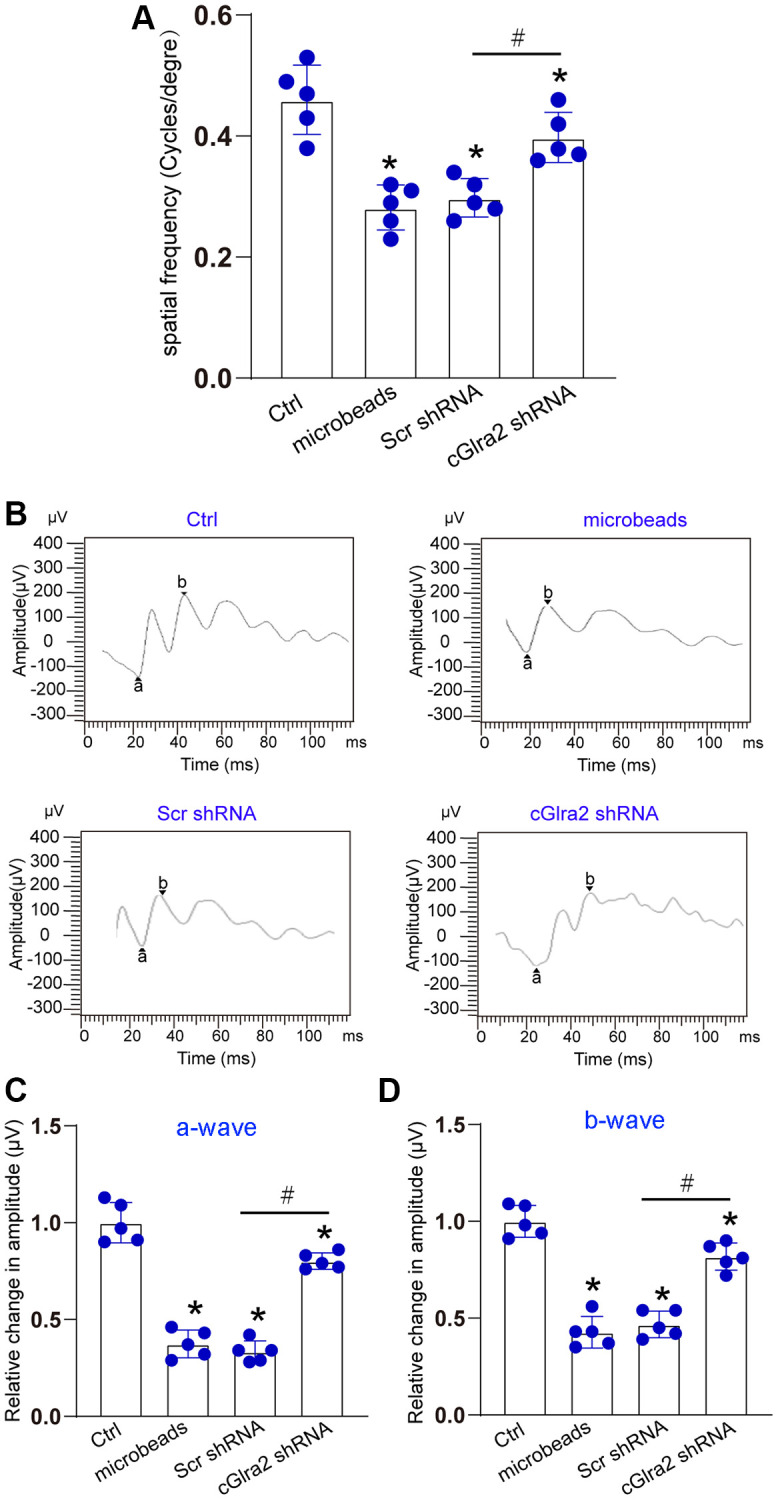
**Silencing of Glra2 alleviates visual acuity and retinal function.** (**A**) Visual acuity was detected by the Cerebral Mechanics OptoMotry virtual-reality optokinetic tracking system. A simple staircase method was employed to detect the highest level of spatial frequency visible to the mouse. All tests were conducted at maximum contrast with a drift speed of 12°/s, starting at a spatial frequency of 0.042 cyc/deg. (**B**–**D**) ERG analysis was performed according to the standard practice and used a 60 Hz low pass digital filter to eliminate the contaminating noises from OPs. The amplitude of a-wave was measured from the baseline to the most negative trough, whereas b-wave amplitude was measured from the trough of a-wave to the highest positive peak. The representative waves are shown in Figure 8B. The amplitudes of a-wave and b-wave were statistically calculated. ^*^P < 0.05 vs. Ctrl; ^#^*P* < 0.05 between the marked groups. The significant difference was evaluated by one-way ANOVA followed by the post hoc Bonferroni test.

The visual acuity of each group was measured by a simple staircase method to determine the highest level of spatial frequency visible to each mouse. Compared with control group, ocular hypertension led to a marked reduction of visual acuity. Injection of cGlra2 shRNA but not scrambled shRNA partially reversed the reduction of visual acuity induced by ocular hypertension (Figure 8A). Electroretinography assays were conducted to detect retinal function. The results showed that ocular hypertension induced by microbeads-injection led to reduced amplitude of a-wave and b-wave. Injection of cGlra2 shRNA but not scrambled shRNA reversed ocular hypertension-induced reduction of the amplitude of a-wave and b- wave as shown by greater a-wave and b-wave amplitude compared with microbeads-injected group, suggesting that cGlra2 silencing alleviates retinal dysfunction induced by ocular hypertension (Figure 8B–8D).

### cGlra2 acts as a miRNA sponge in RGCs

**Figure 9:**
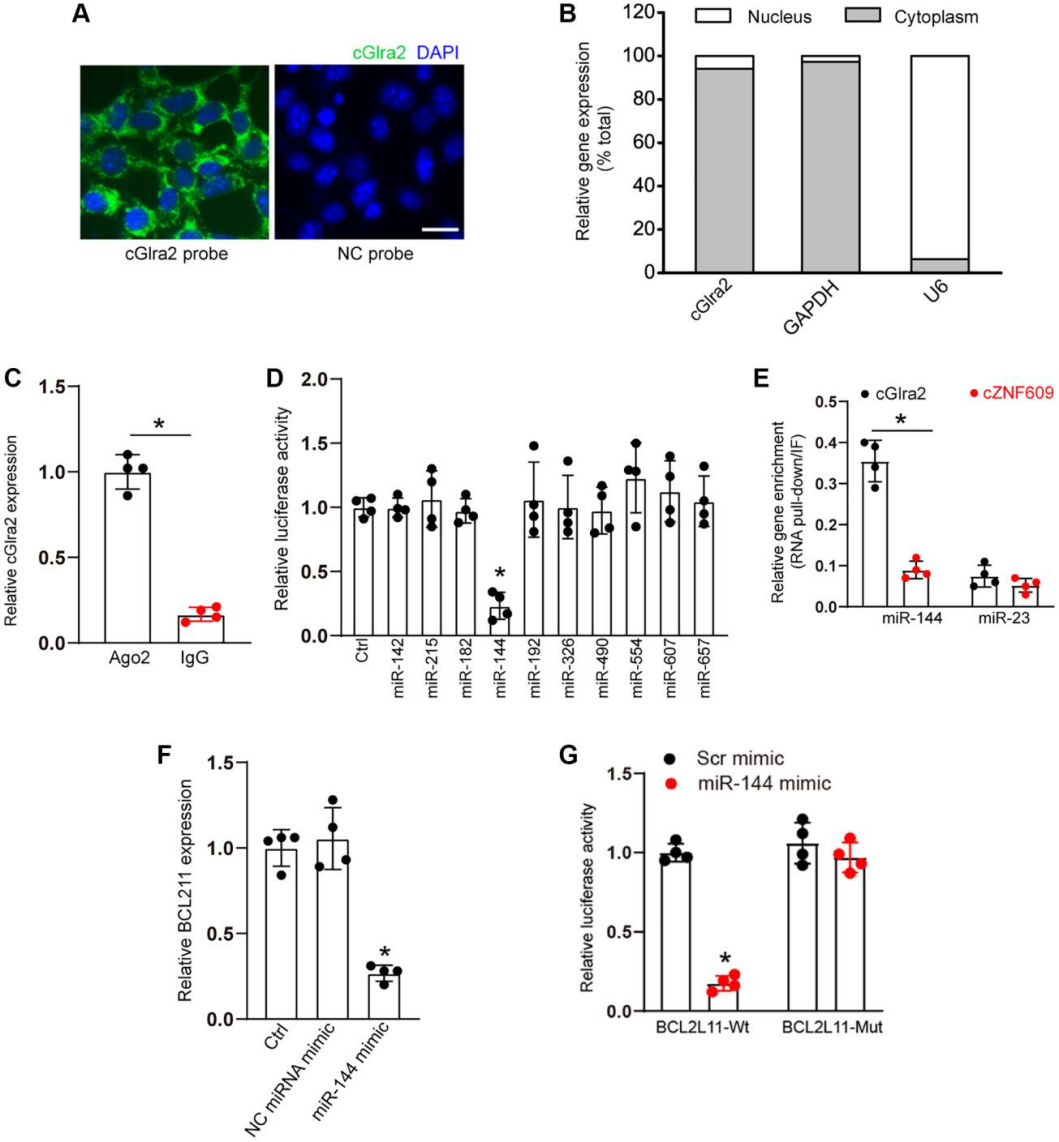
**cGlra2 acts as a miRNA sponge in RGCs.** (**A**, **B**) FISH assays (**A**) and qRT-PCR assays (**B**) were conducted to detect the cytoplasmic and nucleus expression of cGlra2 in RGCs. Scale bar: 20 μm. (**C**) The cytoplasmic fractions of RGCs were immunoprecipitated by Ago2 antibody or IgG. The amount of cGlra2 in RGCs were determined by qRT-PCRs (*n* = 4, *P* < 0.05). (**D**) The entire cGlra2 sequence was cloned into *pGL3* Luciferase Reporter to build Luc-cGlra2 vector. RGCs were co-transfected Luc-cGlra2 with different miRNA mimics. Luciferase activity was determined by the dual luciferase assay following 48-h transfection (*n* = 4, *P* < 0.05). (**E**) The 3’-end biotinylated miR-144 or miR-23 were transfected into RGCs. Following streptavidin capture, the amount of cGlra2 or cZNF609 in bound fractions were determined by qRT-PCR assays. Relative immunoprecipitate (IP)/input ratios were plotted (*n* = 4, *P* < 0.05). (**F**) RGCs were transfected with miR-144 mimics, scramble (Scr) miRNA mimics, or left untreated (Ctrl) for 24 h. qRT-PCRs were conducted to detect the levels of BCL211 expression (*n* = 4, ^*^P < 0.05). (**G**) RGCs were co-transfected LUC-BCL211 with or without miR-144 mimics or Scr miRNA mimics. Luciferase activity was determined by the dual luciferase assays at 24 h post transfection (*n* = 4, ^*^P < 0.05).

qRT-PCR assays and FISH assays revealed that cGlra2 was mainly expressed in the cytoplasm of RGCs (Figure 9A, 9B). Ago2 protein is a crucial component of RNA-induced silencing complex, which can bind miRNAs for mRNA binding [2]. RNA immunoprecipitation assays revealed that cGlra2 was highly enriched the immunoprecipitates pulled down by Ago2 antibody but not IgG (Figure 9C), suggesting that cGlra2 may regulate RGC function by acting as a miRNA sponge. Based on the TargetScan program, we predicted the potential miRNAs which could bind to the sequence of cGlra2. Luciferase activity assays demonstrated that transfection of miR-144 mimic but not other miRNA mimics led to a marked reduction of luciferase activity of LUC-cGlra2 (Figure 9D). We further employed RNA pull down assays to verify the interaction between cGlra2 and miR-144. The result showed that cGlra2 was highly enriched in miR-144-captured fraction compared with the control, biotinylated miR-23 (Figure 9E). We also employed the Targetscan database (https://www.targetscan.org/) to predict the potential target genes of miR-144. One candidate gene, BCL211, aroused our interest due to its role in cell apoptosis. Transfection of miR-144 mimic led to a marked reduction of BCL211 expression (Figure 9F). Luciferase activity assays showed that transfection of miR-144 mimic significantly reduced the luciferase activity of wide-type Luc-BCL211 but had no effect on the luciferase activity of the mutant Luc-BCL211 (Figure 9G). Collectively, the above-mentioned results suggest that cGlra2 acts as a miRNA sponge in RGCs by binding miR-144.

### cGlra2/miR-144/BCL2L11 signaling axis is involved in regulating RGC function

**Figure 10:**
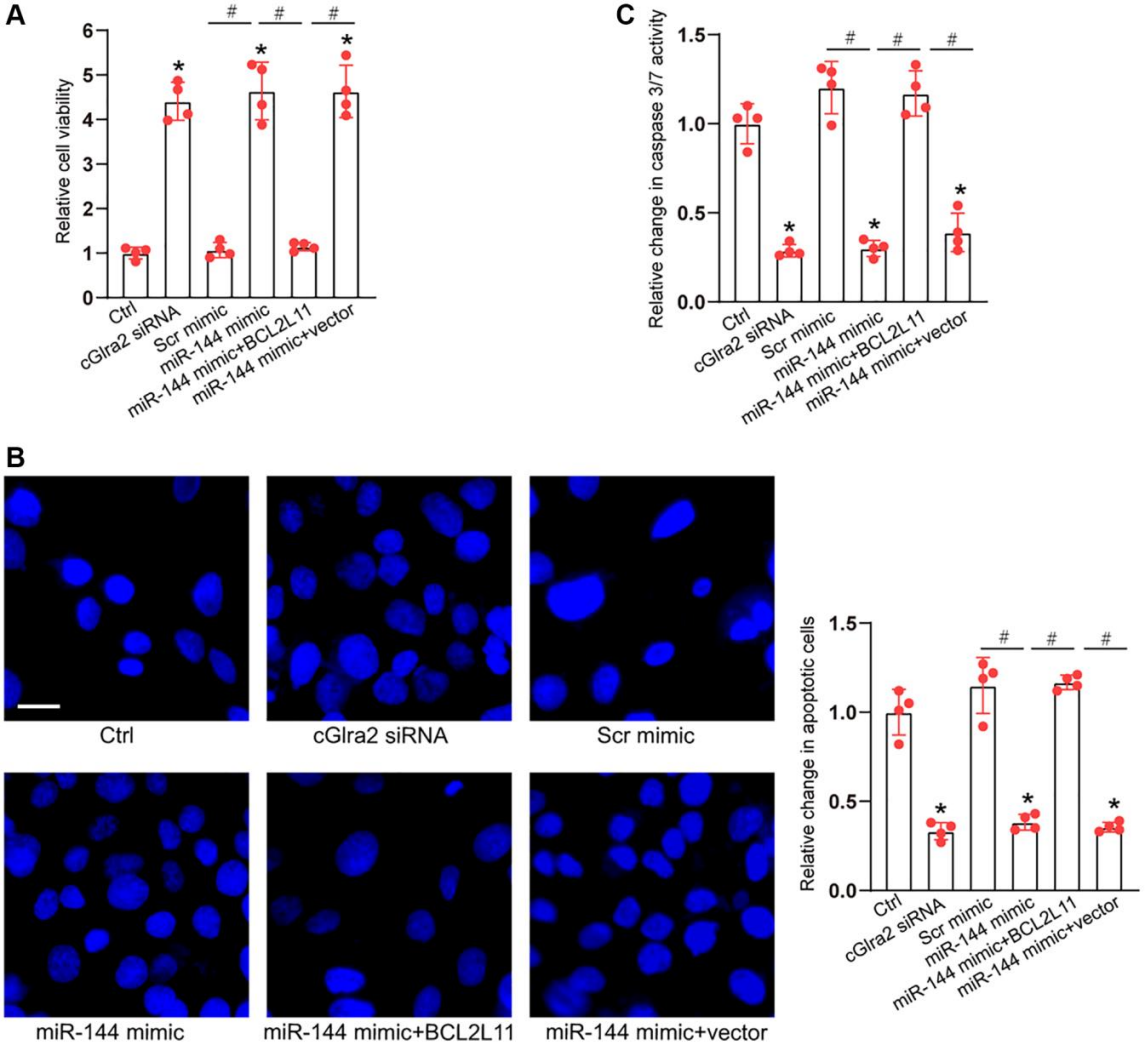
**cGlra2/miR-144/BCL2L11 signaling axis is involved in regulating RGC function.** (**A**–**C**) RGCs were transfected with Scr siRNA, cGlra2 siRNA, scramble (Scr) mimics, miR-144 mimics, miR-144 mimics plus pcDNA3.1-BCL2L11, miR-144 mimics plus pcDNA3.1 (vector) or left untreated (Ctrl) for 12 h and then exposed to H_2_O_2_ (100 μmol/L) to mimic oxidative stress for additional 36 h. CCK-8 assays were performed to detect RGC viability (**A**, *n* = 4). Hoechst staining and quantification analysis was performed to detect the change of nuclei morphological characteristics of RGCs (**B**, *n* = 4, Scale bar: 50 μm). Caspase 3/7 activity was performed to detect the degree of RGC apoptosis (**C**, *n* = 4). ^*^P < 0.05 vs. Ctrl group; ^#^*P* < 0.05 between the marked group. All significance was examined using One-way ANOVA followed by Bonferroni’s post hoc test.

As miR-144 was sponged by cGlra2, we then investigated the role of miR-144 in RGC biology. Under oxidative stress, CCK-8 assays demonstrated that compared with Ctrl group (H_2_O_2_ treatment alone), transfection of miR-144 mimics significantly enhanced the viability of RGCs and alleviated oxidative stress-induced reduction of RGC viability, which could mimic the effects of cGlra2 silencing on RGC function. By contrast, overexpression of BCL2L11 but not null vector could interrupt the effects of miR-144 mimic on RGC viability (Figure 10A). Hoechst staining assays showed that compare with Ctrl group (H_2_O_2_ treatment alone), transfection of miR-144 mimics decreased the number of condensate and fragmented nuclei in RGCs, mimicking the effects of cGlra2 silencing, while overexpression of BCL2L11 could interrupt the protective effects of miR-144 mimics on RGC function (Figure 10B). Caspase 3/7 activity assays demonstrated that transfection of miR-144 mimics significantly reduced the activity of caspase 3/7, showing a similar effect as cGlra2 silencing. By contrast, overexpression of BCL2L11 interrupted the effects of miR-144 mimics on RGCs (Figure 10C). Under hydrostatic stress, transfection of miR-144 mimic alleviated hydrostatic stress-induced reduction of RGC viability, decreased the number of condensate and fragmented nuclei, and reduced the activity of caspase 3/7, showing a similar effect as cGlra2 silencing. By contrast, overexpression of BCL2L11 could interrupt the effects of miR-144 mimics on RGC functions (Supplementary Figure 5).

## DISCUSSION

Glaucoma is a major cause of irreversible blindness in the world. Lowering IOP level can reduce the progressive loss of visual field. However, there are still some patients suffering from visual loss following lowering IOP [15]. Thus, further studies are still required to clarify the pathogenesis of glaucoma. In this study, we determined circRNA expression profiling and investigated the role of circRNA in glaucomatous neurodegeneration. The results revealed that 101 circRNAs were significantly up-regulated and 70 circRNAs were significantly down-regulated in the glaucomatous retinas. We further conducted qRT-PCR assays to verify the results of circRNA microarray. The expression pattern of selected circRNAs was confirmed by qRT-PCRs, suggesting that the result of circRNA microarray was credible. This finding can provide an important theoretical basis for clarifying the role of circRNAs in glaucomatous neurodegeneration.

circRNAs can modulate gene expression in several biological processes, including neural development, neural differentiation, and neurodegenerative process. Retina is a neural tissue and shares several common features with the central nervous system. Clarifying the mechanism of retinal neurodegeneration can provide novel insights into neurodegenerative diseases [10]. Previous studies have shown that silencing of circRNA-ZYG11B exerts a neuroprotective effect against ischemic retinal diseases. cZNF609 expression is obviously induced during retinal neurodegeneration and silencing of cZNF609 can reduce retinal reactive gliosis and facilitate RGC survival. cZRANB1 expression is significantly up-regulated during retinal neurodegeneration. Targeting cZRANB1 is an effective strategy for treating retinal neurodegeneration [2, 11, 12]. In this study, we reveal that silencing of cGlra2 can alleviate RGC apoptosis, decrease reactive gliosis, and reduce retinal cell apoptosis. Moreover, detecting the level of cGlra2 in aqueous humor makes it possible to distinguish glaucoma patients from cataract patients. circRNA are often generated from their parental genes and interact with each other to regulate biological process [16]. We thus performed GO enrichment analysis and pathway enrichment analysis to predict the potential role of circRNA-mediated signaling. The most enriched CC terms are related to cytosol, suggesting the dysregulated circRNAs-mediated signaling mainly occurs at the post-transcriptional level. ATP binding is shown as the most enriched and meaningful MF term. ATP binding is associated with energy metabolism. Thus, glaucoma is tightly associated with metabolic condition. Synaptic transmission is the most enriched BP term. Abnormal synaptic transmission has been implicated in the pathogenesis of glaucoma [17, 18]. KEGG analysis reveals that the most enriched signaling pathway involved in the dysregulated circRNA-mediated signaling is MAPK signaling, which has been reported to regulate RGC function and retinal neurodegeneration [19, 20]. Based on the above-mentioned results, we speculate that circRNA-mediated signaling is tightly associated with the pathogenesis of glaucoma.

AH is actively secreted in non-pigmented ciliary epithelium and diffused across iris root [21]. We collected AH samples from glaucomatous eyes and cataract eyes. Detection the level of cGlra2 in AH samples can discriminate glaucoma patients from cataract patients. The origin of circRNAs in AH samples is still unknown. circRNAs in AH samples may be affected by the breakdown of anterior ocular segment, breakdown of posterior segment, or cell fragments [22, 23]. Thus, the change of AH constitutes occurs during the pathological processes of ocular diseases. The levels of cGlra2 are up-regulated in AH samples of glaucoma patients. Increased cGlra2 may aggravate RGC degeneration and accelerate retinal neurodegeneration.

circRNAs can regulate gene expression at the post-transcriptional level. They are involved in several biological processes such as cellular growth, differentiation, and cell death [24]. RGC degeneration is the final common pathway in both human and animal models of glaucoma [1]. In this study, we show that silencing of cGlra2 protects RGCs against oxidative stress- or hydrostatic stress-induced injuries. Moreover, silencing of cGlra2 can alleviate ocular hypertension-induced retinal neurodegeneration *in vivo*. RGCs are known as the major cells, which can execute the neural functions in visual pathway. Glial cells can support neural homeostasis, regulate neurotransmitter scavenging, and affect energy supply [25]. Abnormal reactive gliosis can cause exacerbated neuronal degeneration and increased retinal apoptosis. Silencing of cGlra2 can alleviate glaucomatous neurodegeneration as shown by increased TUJ1 staining and reduced retinal reactive gliosis and retinal apoptosis. Thus, silencing of cGlra2 is a potential method for treating retinal neurodegeneration.

BCL2L11 (also known as BIM) is a BH3-only protein, which can induce apoptosis by inactivating anti-apoptotic BCL2 proteins or activating BAX-BAK1 [26]. Increased BCL2L11 expression can indicate the pathway to retinal cell death in development and degeneration. Under high glucose condition, increased Bim expression contributes to the apoptosis of retinal pericytes [27, 28]. Bim has been involved in external injuries-induced retinal ganglion cell death following optic nerve transection [27]. This evidence suggests that BCL2L11 is a critical regulator of retinal neurodegeneration. In this study, we show that transfection of miR-144 mimic alleviates hydrostatic stress and oxidative stress-induced reduction of RGC viability, decreases the number of the condensate and fragmented nuclei, and reduces the activity of caspase 3/7, showing a similar effect as cGlra2 silencing. By contrast, overexpression of BCL2L11 can interrupt the effects of miR-144 mimics on RGC functions. During RGC degeneration, cGlra2 overexpression becomes a sink of miR-144 and release the inhibitory effects of miR-144 on BCL2L11. BCL2L11 up-regulation contributes to RGC injuries during retinal degeneration.

In conclusion, this study reveals that circRNA expression pattern in the glaucomatous retinas is significantly different from that in normal retinas. Silencing of cGlra2 can protect against oxidative stress and hydrostatic stress-induced RGC injury and retard glaucomatous neurodegeneration. The preliminary observation demonstrates that cGlra2 level in AH sample can be used as a biomarker of glaucoma. Detection of the levels of cGlra2 in the clinical samples can discriminate glaucoma patients from cataract patients. Further studies in determining the role of circRNAs in glaucoma may help design novel strategies for the diagnosis and treatment of glaucoma.

In this study, we identified the differentially expressed circRNAs in a murine model of retinal neurodegeneration and clarified the role of cGlra2 in retinal neurodegeneration *in vivo* and *in vitro*, but there were still a number of limitations. First, we have recruited as many glaucoma patients as possible to evaluate the specificity and sensitivity of cGlra2 for glaucoma diagnosis, but the sample size is still very small. Second, it is still challenging to knockout a gene especially a circRNA in a specific retinal cell type. Non-specific knockdown of cGlra2 not only affects cGlra2 expression in RGCs but also affects cGlra2 expression in other retinal cells *in vivo*. Despite we have verified the role of cGlra2 in RGC degeneration *in vitro*, we cannot rule out the possibility that silencing of cGlra2 can also affect the functions of other retinal cells. Finally, the mechanism of cGlra2-medaited retinal neurodegeneration is still required to be investigated in future study.

## MATERIALS AND METHODS

### Isolation of primary mouse RGCs

The retinas from the postnatal day 3 mice were digested with papain (5 mg/mL) for 45 min at room temperature. The cell suspensions were incubated with the anti-macrophage antibody (Dilution ratio: 1:150; cat. no. AIA31240; Accurate Chemical and Scientific Corporation) to remove macrophages. The non-adherent cells were then transferred to culture plates, which were pre-conjugated with anti-TUJ1 antibody (Dilution ratio: 1:50, ab18207, Abcam, USA) to purify RGCs. The plates were washed with PBS buffer to remove non-adherent cells. The adherent cells were digested with trypsin (1,250 units/mL, T4799, BioFroxx, Germany) and cultured in DMEM medium (8120034, Gibco, USA) plus insulin (1.5 mM), progesterone (30 nM), CNTF (30 ng/ml), and forskolin (3 μM). The identification of RGCs was conducted by staining with TUJ1 (Dilution ratio: 1:200) and Thy 1.2 (Dilution ratio: 1:200).

### Cell transfection

RGCs were seeded onto the 6-well plates (2 × 10^5^ cells/well) at 37°C overnight. After the confluence reached about 85%, they were transfected with cGlra2 siRNA or scrambled siRNA using Lipofectamine 3000 (Invitrogen, USA). siRNA sequences were shown below: cGlra2 siRNA, 5′-AGGUCCUCCAGUUAAACGUUACUUTdTd-3′ (sense); cGlra2 siRNA, 5′-AAGUAACGUUUACUGGAGGACCUTdTd-3′ (anti-sense); scrambled siRNA, 5′-UUCUCCGAACGUGUCACGUTdTd-3′ (sense), 5′-ACGUGACACGUUCGGAGAATdTdT-3′ (anti-sense). siRNA sequences were synthesized by Sangon Biotech Co., Ltd (Shanghai, China). The final concentrations of siRNAs were 30 nM.

### Retinal frozen section for immunohistochemistry

The eyeballs were fixed with 4% paraformaldehyde (PFA) at room temperature for 12 h, immersed into 30% sucrose solution for additional 12 h, and embedded in the optimum cutting temperature compound. Next, 10-μm retinal sections were cut and tiled on gelation-coated slides. After rehydration, retinal slices were blocked with 5% bovine serum albumin (BSA) for 0.5 h at 37°C. The slices were incubated with the primary antibody (Dilution ratio: 1:200) overnight at 4°C, washed with PBS solution containing 0.1% Tween 20, and incubated with secondary antibody (Dilution ratio: 1:1000) overnight at 4°C. Finally, the nuclei were labeled with DAPI for 10 min and observed under a fluorescence microscope [29].

### Hydrostatic pressure experiment

RGCs were cultured at the ambient pressure or hydrostatic pressure to determine the effects of elevated pressures on RGC function [30]. In ambient pressure experiment, RGCs were cultured in a standard incubator. In hydrostatic pressure experiment, RGCs were cultured in a custom-made regulator chamber placed in the incubator. The mixture of 95% air and 5% CO_2_ was pumped into the custom-made chamber to obtain 70 mm Hg pressure.

### Cell counting kit-8 (CCK-8) assay

Cell viability was detected by a CCK-8 kit (Dojindo) according to the manufacturer’s protocols. Briefly, about 3 × 10^3^ of RGCs per well were cultured in a 96-well plate in triplicate. Then, 30 μL of CCK-8 solution was added into each well for 2 h at 37°C. Next, 100 μL of dimethyl sulfoxide (DMSO) was added to each well to solubilize formazan products. Finally, the absorbance was recorded at 450 nm using a microplate reader (Bio-Rad, CA, USA). Relative cell viability was normalized with the control group using optical density value.

### Caspase-3/7 activity assay

The activity of caspase 3/7 was detected by a caspase 3/7 assay kit (Beyotime, Nantong, China). RGCs were incubated with 100 μl of the substrate per well for 45 min. Caspase 3/7 activity was detected at 490 nm excitation wavelength and 525 nm emission wavelength. Each sample was measured in duplicate [31].

### Hoechst staining

The survival state of RGCs was determined by Hoechst 33342 staining (62249, Thermo Fisher Scientific, USA) to show the morphological characteristics of nuclei. After the required treatment, RGCs were fixed in 4% PFA at room temperature for 10 min. Then, RGCs were permeabilized with 0.25% Triton X-100 for 10 min and stained with Hoechst 33342 (100 μg/ml) for 5 min. The signal of stained cells was observed under a fluorescence microscope with an excitation wavelength at 359 nm and an emission wavelength at 461 nm.

### JC-1 staining

Mitochondrial membrane potential (ΔΨm) was detected by 5,5′,6,6′-tetrachloro-1,1′,3,3′-tetraethyl-benzimidazolyl-carbocyanine iodide or J-aggregate-forming cation (JC-1) dye (ab113850, Abcam, USA). After 48 h incubation, RGCs were stained with JC-1 (2 μg/ml) for 15 min, washed twice with PBS, and observed under a fluorescence microscope. Red fluorescence was indicative of intact mitochondria, while green fluorescence was indicative of loss in ΔΨm [32].

### Retinal whole flat-mount staining

The retinas were fixed in 4% PFA for 30 min and dissected into the petal shape as the whole flat-mount. The retinas were blocked with 5% BSA for 60 min, incubated with anti-TUJ1 antibody (Dilution ratio: 1:200, Biolegend, 801201, USA) overnight at 4°C. Then, the retinas were washed with PBS buffer and incubated with the Alexa Fluor 594-conjugated goat anti-mouse IgG (Dilution ratio: 1:500, Invitrogen, USA) for 3 h at room temperature. TUJ1-positive cells were counted using the ImagePro Plus 6.0 (Media Cybernetics, USA) software. RGC survival rate was evaluated by dividing the average number of TUJ1-positive cells in the injured retinas by that in normal retinas.

### Visual acuity

The virtual-reality optokinetic tracking system (OptoMotry, Canada) was used to detect visual acuity by an optokinetic head-tracking (OKT) test [33]. Briefly, the mice were placed on a pedestal inside a chamber consisting of 4 monitors. The mice were allowed to move freely. An alternating rotating stimulus was shown on the screen in three-dimensional space. These mice tracked the stimulus until they could not see. The tester recorded the presence and absence of tracking and a simple staircase method was employed to determine the highest level of spatial frequency visible to mice. The tests were conducted at the maximum contrast with a drift speed of 12°/s and a spatial frequency of 0.042 cyc/deg.

### Electroretinography

After an overnight dark adaptation, the mice were anaesthetized and the pupils were dilated with 1% tropicamide. Then, the mice were positioned on a heating pad. ERGs were recorded with a gold electrode inserted in corneal lens (LKC Technologies, USA). The reference and ground electrodes were inserted in the forehead and in the tail. The signals were acquired using the software Signal (v.3.01x, CED, Cambridge UK). ERG analysis was performed according to standard practice and used a 60 Hz low pass digital filter to eliminate the noises from OPs. The amplitudes of a-wave were measured from the baseline to the most negative trough, whereas b-wave amplitudes were measured from the trough of a-wave to the highest positive peak.

### Optic nerve histopathology

Following the required treatments, the optic nerves were isolated and 5-μm longitudinal paraffin embedded sections were prepared. Longitudinal optic nerve sections were stained by hematoxylin and eosin (H&E). Three images were taken at 40 × magnification of each stained nerve (one each of the proximal, central, and distal portion of the optic nerve). The swellings of RGC axons were calculated using ImagePro Plus 6.0 software (Media Cybernetics, USA).

### TUNEL staining

The eyeballs were fixed in 4% PFA at room temperature for 12 h, immersed in 30% sucrose solution for 12 h, and embedded in optimum cutting temperature compound. The retinas were then enucleated and sectioned at 10-μm thickness using a cryostat (Leica CM1850). For each eye, 3–4 serial sections were taken with a step of 50 μm. The apoptosis of retinal sections was detected by a TUNEL Apoptosis Detection Kit (Ribobio, Guangzhou, China) according to the manufacturer’s protocols. Briefly, retinal sections were incubated with the blocking buffer (3% H_2_O_2_ in CH_3_OH) for 30 min at room temperature. After washing with PBS three times, retinal sections were incubated with TUNEL reaction mixture for 1 h at 37°C. Following washing with PBS three times, DAPI was added for 1 min to label nuclei. The slides were mounted and examined by a fluorescence microscope.

### Quantitative RT-PCR

Total RNAs were isolated from the retinas or RGCs using a Qiagen RNeasy Mini Kit. Briefly, the isolated RNAs were treated with DNase (Invitrogen, USA) to ensure no contamination of genomic DNAs. About 500 ng of total RNAs were reversely transcribed (Thermo Fisher Scientific, USA). Quantitative PCRs were performed in a 25 μl volume using the FastStart Universal SYBR Green Master (Sigma-Aldrich, USA), which were performed as shown below: denaturation at 95°C for 5 min, followed by 45 cycles of 95°C for 10 sec and 64°C for 45 sec. Relative gene expression was determined using the 2^−ΔΔCt^ methods. The primer sequences used in this study are shown in Table 1.

**Table 1:** Primer sequences used for qPCR assays.

mmu_circ_0016226	Forward primer	5′-CTGGGCATCACAACAGTCCT-3′
	Reverse primer	5′-CCAGCCGTGAATCATTCCAC-3′
mmu_circ_0016224	Forward primer	5′-TCCTTCTGTGGGTTGTAAATTCT-3′
	Reverse primer	5′-CCATTGAGAACTTGACCTTGCA-3′
cicRNA.16113	Forward primer	5′-AGCCACAGACTTATTTTCATGCT-3′
	Reverse primer	5′-GCCAGGCCCCAAACATTTTA-3′
cicRNA.7215	Forward primer	5′-GGTGACCATTCCTTCTCTCCA-3′
	Reverse primer	5′-AAACTCACTCCCCTCCAGAC-3′
cicRNA.27992	Forward primer	5′-TCAGTACAAACCAATGAAGCCA-3′
	Reverse primer	5′-GCCTGGATGCAGTGTTGAAA-3′
mmu_circ_0016436	Forward primer	5′-GCAGCAGGGAAAGAGGTTTT-3′
	Reverse primer	5′-TCCCTGCTGATACAACACGT-3′
hsa_circ_0139862	Forward primer	5′-ATGACCACCCAGAGTTCAGG-3′
	Reverse primer	5′-CTCGGTAGTCCATGGTCGTT-3′

### High IOP mouse model

Retinal neurodegeneration was induced by enhanced IOP using 8-week-old C57BL/6J mice. In brief, the mice were anesthetized with the mixture of ketamine/xylazine (100 mg/kg and 20 mg/kg). After dilation using topical tropicamide-phenylephrine hydrochloride (Santen), a 31-gauge needle was used to make a corneal incision penetrated the whole cornea from the temporal to superior cornea. The glass micropipette (outer diameter: 1.0 mm; inner diameter: 0.75 mm) connected to a Hamilton syringe (25 μL) by polyethylene tube (0.86 mm). A 20-gauge needle was inserted into the anterior chamber for the injection of microbead suspensions. The microbead suspensions were prepared as follows. The stock suspension of FluoSphere Polystyrene Microspheres (15 μM diameter, Life Technologies, USA) was left overnight to segregate suspension. After removing the supernatant, the beads were re-suspended in saline solution. One eye was injected with microbead suspension (3.0 × 10^7^ beads/mL, 2.0 μL) and the other eye was injected with the same volume of saline suspension. Another injection of beads or saline solution was required 4-weeks following initial injection. IOP measurement was performed using a TonoLab tonometer (Colonial Medical Supply, USA) twice weekly in both eyes for 8 weeks. After general anesthesia, the mice were placed on a platform and the tip of probe was kept on the central area of cornea. IOP measurements were conducted between 10 AM and 12 PM to minimize the variability. The result of each measurement was an average of three measurements and the average of three such readings (9 measurements) was considered as IOP value [3, 23].

### circRNA microarray profiling

The samples were collected from microbeads-injected retinas (*n* = 12) and saline-injected retinas (*n* = 12) at 2-months following injection. RNA samples from 4 different retinas were pooled together as a biological replicate. The linear RNAs were degraded with RNase R to enrich circRNAs and transcribed into the fluorescent cRNAs. cRNAs probes were purified by a RNeasy Mini Kit (Qiagen, Germany). CircRNA expression profiling was performed by the Arraystar Mouse circRNA Array V2. Gene Expression Hybridization Kit (Agilent Technologies, USA) was employed for the hybridization. The labeled cRNAs were incubated with the blocking agent and the fragmentation buffer for fragment. Then, the labeled cRNAs were diluted. The hybridization solutions were dispensed into the gasket slide and assembled onto circRNA expression microarray slide. Finally, the slides were heated and Agilent Microarray Scanner (Agilent Technologies, USA) was used to obtain circRNA microarray signaling. Microarray signaling was extracted using the Agilent Feature Extraction software. Microarray analysis was performed via *R* software package. Differential expressed circRNAs were identified using volcano plot and hierarchical clustering [29].

### Gene ontology and KEGG pathway analysis of parental genes of dysregulated circRNAs

The functions of parental genes of the dysregulated circRNAs were predicted by Gene ontology (GO) enrichment analysis and KEGG pathway analysis (http://www.genome.jp/kegg/). GO enrichment analysis revealed enriched biological processes (BP), cellular components (CC), and molecular function (MF) of parental genes of the dysregulated circRNAs. KEGG analysis revealed the enriched signaling of parental genes of the dysregulated circRNAs during retinal neurodegeneration [34, 35].

### Fluorescence *in situ* hybridization (FISH) assay

FISH assays were conducted using the Cy3-labelled cGlra2 probe and negative control probe. The Glra2 probe sequence: TCAGAAAAATGTTCACTCGGTAGTCCTTTGGCAGAGATGCCCTGGAACCT. The control probe sequence: AAACAGTACTGGTGTGTAGTACGAGCTGAAGCTAC. Briefly, retinal sections were fixed in 4% PFA for 1 h. Following fixation and washing with PBS, retinal sections were permeabilized with 0.5% Triton X-100 for 10 min and pre-hybridized with the pre-hybridization buffer (contain 1% blocking solution) for 30 min at 37°C. After washing with PBS three times for 5 min each time, retinal sections were incubated with the fluorescence probes in the hybridization buffer (40% formamide, 10% Dextran sulfate, 1 × Denhardt’s solution, 4 × SSC, 10 mM DDT, 1 mg ml^−1^ yeast transfer RNA, 1 mg ml^−1^ sheared salmon sperm DNA) at 37°C for 6 h. Afterwards, they were washed in the gradient decreased concentration of SSC buffer at 42°C (4 × SSC buffer for 5 min trice, 2 × SSC buffer for 5 min, 1 × SSC buffer for 5 min). Finally, they were incubated with 4, 6-diamidino-2-phenylindole (DAPI, D1306) for 10 min at room temperature to label the nuclei and mounted with the anti-fade fluorescent mounting medium (Dako).

### Production and transduction of cGlra2 shRNA adeno-associated virus

About 200 pmol of the forward and reverse primers containing cGlra2 shRNA sequence or scramble shRNA sequence were dissolved in 2 × annealing buffer (20 mM Tris, pH 7.8, 100 mM NaCl and 0.2 mM EDTA). The solution was boiled for 10 min and cooled. The annealed oligos were ligated and cloned into AAV2 vector. For AAV shRNA package, these vectors were co-transfected with pAAV-RC1 and pHelper vector into HEK293T packaging cell line. Forty-eight hours following transfection, these cells were harvested and purified by ultracentrifugation. Vector preparations were purified by dialysis and titered by qRT-PCRs.

The mice were anesthetized by the mixture of ketamine (100 mg/kg) and xylazine (10 mg/kg). The pupils were dilated with 0.5% tropicamide. About 1 μL (1 × 10^10^ viral particles/mL) of adeno-associated virus 2 (AAV2) containing cGlra2 shRNA or scramble shRNA was delivered into vitreous cavity through corneal and scleral limbus for cGlra2 silencing using a Hamilton syringe with 33-gauge disposable needle. The ocular surfaces were covered with levofloxacin hydrochloride eye gel to prevent infection and efflux. The mice were kept warm at 37°C until anesthesia emerged.

### Biotin-coupled miRNA capture

The 3′-end biotinylated miR-144 or control miR-23 were transfected into RGCs for 24 h. The biotin-coupled RNA complexes were immunoprecipitated by incubating the lysates with the streptavidin-coated magnetic beads (Life Technologies, USA). The amounts of cGlra2 and cZNF609 in the bound fractions were detected by qRT-PCR assays [36].

### Cytoplasm/nucleus fraction isolation

Cytoplasm/nucleus fraction ratio was determined by a Nuclear/Cytosol Fractionation Kit (Cell Biolabs, USA). Briefly, RNAs were extracted from the cytoplasm or nucleus fractions of RGCs. Relative levels of cGlra2, nucleus control transcript (U6), and cytoplasmic control transcript (GAPDH) were detected by qRT-PCR assays [37].

### Luciferase assay

Luciferase assays were performed by a luciferase assay kit (Promega, USA) according to the manufacturer’s protocols. Briefly, RGCs were seeded in 24-well plates at about 85% confluence. The entire cGlra2 sequence was cloned into the pGL3 Luciferase Reporter to build Luc-cGlra2 vector. The wild-type or mutant 3′-untranslated region (UTR) of BCL211 was constructed and cloned into the pGL3 Luciferase Reporter. RGCs were then co-transfected with miRNA mimics using Lipofectamine 6000 (Beyotime Technology, China). After 48 h post transfection, the activities of *Firefly* and *Renilla* luciferase were determined by a luciferase assay kit.

### RNA immunoprecipitation assay (RIP)

RIP assays were conducted using as an EZ-Magna RIP™-Binding Protein Immunoprecipitation Kit (Millipore, USA) according to the manufacturer’s instruction. RGCs were lysed in RIP lysis buffer (20 mM Tris-CL, pH 7.5, 150 mM NaCl, 1 mM ethylenediaminetetraacetic acid, 0.5% NP-40, and 5 μg/ml aprotinin), and then incubated with anti-IgG antibody (negative control) or anti-Ago2 antibody at 4°C for 6 h. Protein A-Sepharose was added to each sample and the mixtures were incubated at 4°C for 3 h. The pellets were washed with PBS and re-suspended in 0.5 ml of TriReagent (Sigma-Aldrich, USA). The precipitated RNAs in aqueous solutions were subject for qRT-PCR assays [12].

### Clinical sample collection

Aqueous humor (AH) samples were obtained between May 2018 and May 2020. During AH collection, all patients received topical anesthesia with 0.5% proparacaine hydrochloride (Alcon Pharmaceuticals) drops applied to ocular surface before injection. Following the patients being sterilized with betadine, the surgeon punctured the corneas with a 26 gauge-needle and AH samples were aspirated until the iris came in front and contracted. Great care should be taken to avoid touching the lens. Topical antibiotic was applied again after AH collection.

AH samples were obtained from the patients, which were diagnosed as acute angle-closure glaucoma (AACG). AACG requiring the paracentesis of anterior chamber was included when there were the symptoms and signs of sudden increase of IOP level (IOP ≥21 mmHg), ocular pain, halo, and corneal edema. The diagnosis of AACG also needed the presence of appositional angle closure over 270° or more and the corresponding glaucomatous optic neuropathy. Exclusion criteria were shown below: previous intraocular surgery, including cataract, glaucoma, and laser peripheral iridotomy; history of anti-VEGF treatment; patients with lens subluxation or intumescent cataract; patients with uveal effusion or uveitis; patients with retinal detachment or vitreoretinal traction; patients with axial lengths <19 mm in either eye. The control samples were collected from patients diagnosed with cataract.

### Data analysis

All data were shown as mean ± SD. The statistical difference was determined by Student *t*-test or one-way ANOVA. The result was considered to be statistically different when *P* < 0.05.

### Availability of data and materials

The datasets generated and/or analyzed in the current study are available from the corresponding author upon reasonable request.

## Supplementary Materials

Supplementary Figures

Supplementary Table 1

Supplementary Table 2
