# Supplementary material for: Targeting circular RNA-Glra2 alleviates retinal neurodegeneration induced by ocular hypertension
Source: Aging (Albany NY). 2023 Oct 10;15(19):10705–31. doi: 10.18632/aging.205108 (PMC10599745; doi:10.18632/aging.205108)
Supplement: Supplementary Figures [file aging-15-205108-s001.pdf]

SUPPLEMENTARY FIGURES

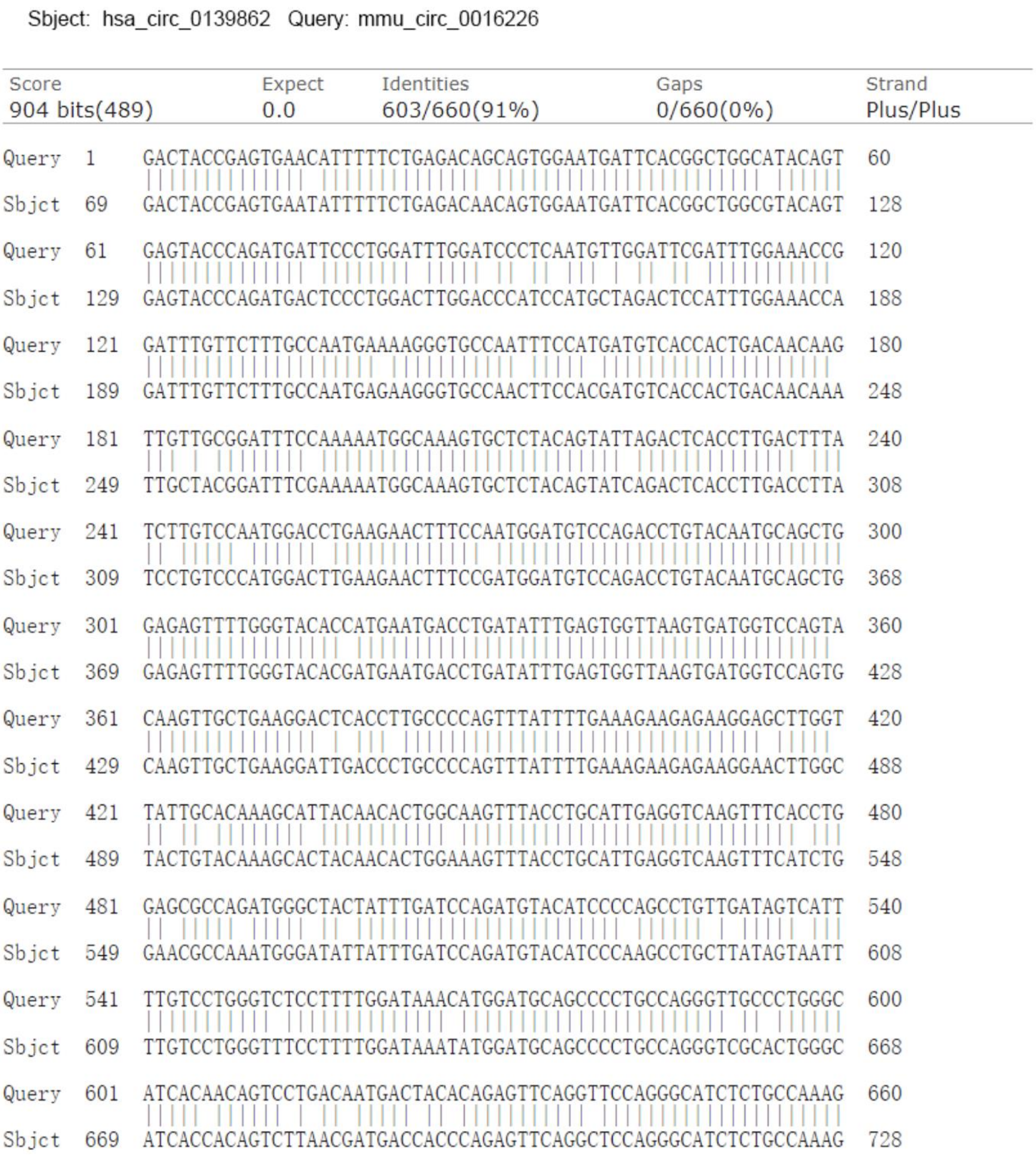

Supplementary Figure 1. Homologous gene comparison of cGlra2. NCBI BLAST was conducted to compare the sequence similarity of cGlra2 between human genome and murine genome.

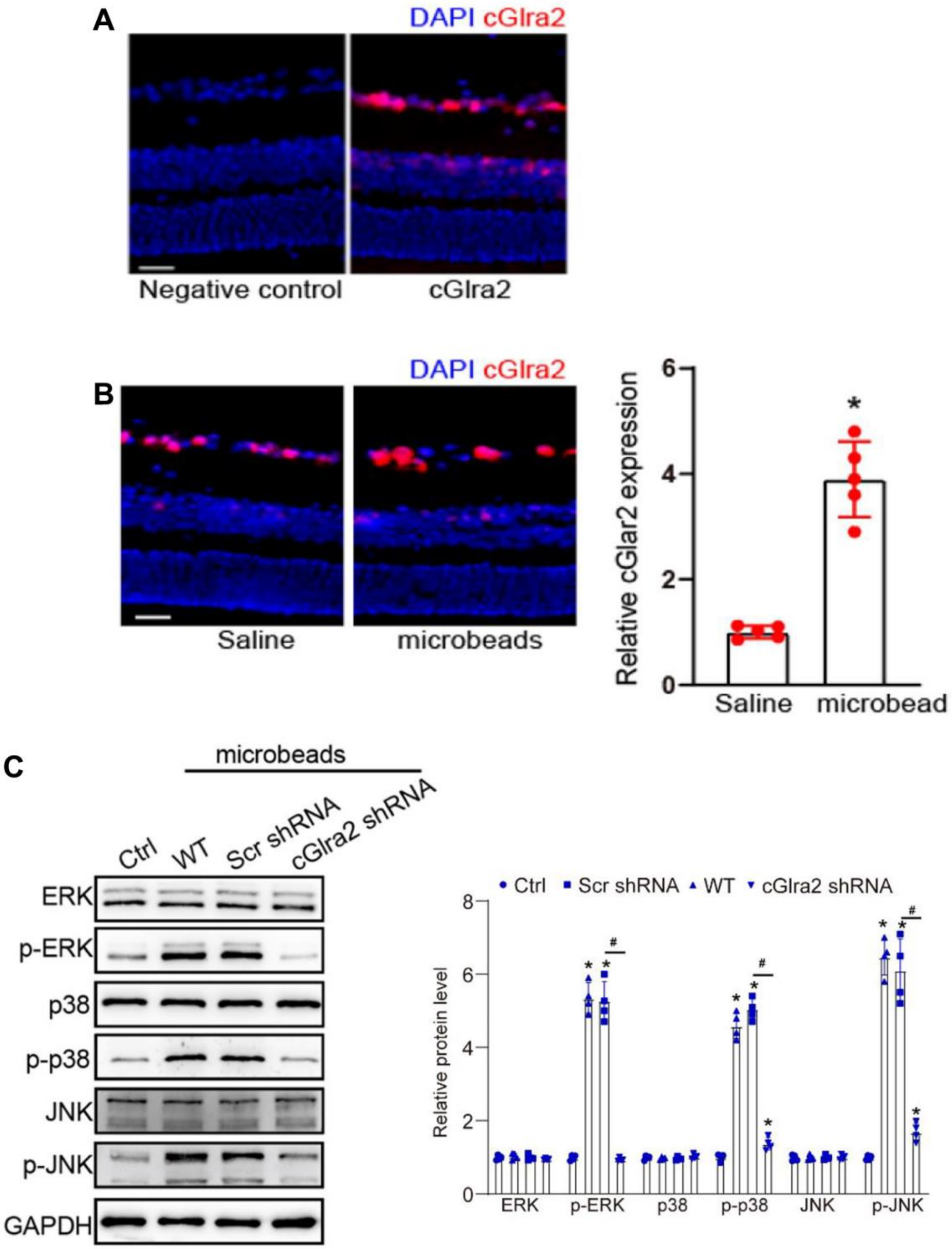

Supplementary Figure 2. Detection of cGlra2 expression in retinal tissues. (**A**) The expression distribution of cGlra2 in retinal tissues was detected by FISH assays. Red fluorescence represents the expression pattern of cGlra2 and blue fluorescence is DAPI. Scale bar: 20 µm. (**B**) FISH assays were conducted to compare the expression pattern of cGlra2 between the microbeads-injected retinas and the saline-injected retinas. Scale bar: 20 µm. *n* = 5 animals. (**C**) Western blots were conducted to detect the expression levels of the members of MAPK signaling in saline-injected retinas (Ctrl), microbeads-injected retinas (WT), microbeads-injected retinas plus Scr shRNA, and microbeads-injected retinas plus cGlra2 shRNA following 2-month after the induction of ocular hypertension. GAPDH was detected as the internal control (*n* = 4 animals; $^{*}P < 0.05$ vs. saline-injected retinas; $^{\#}P < 0.05$ between the marked group; One-way ANOVA followed by Bonferroni's post hoc test).

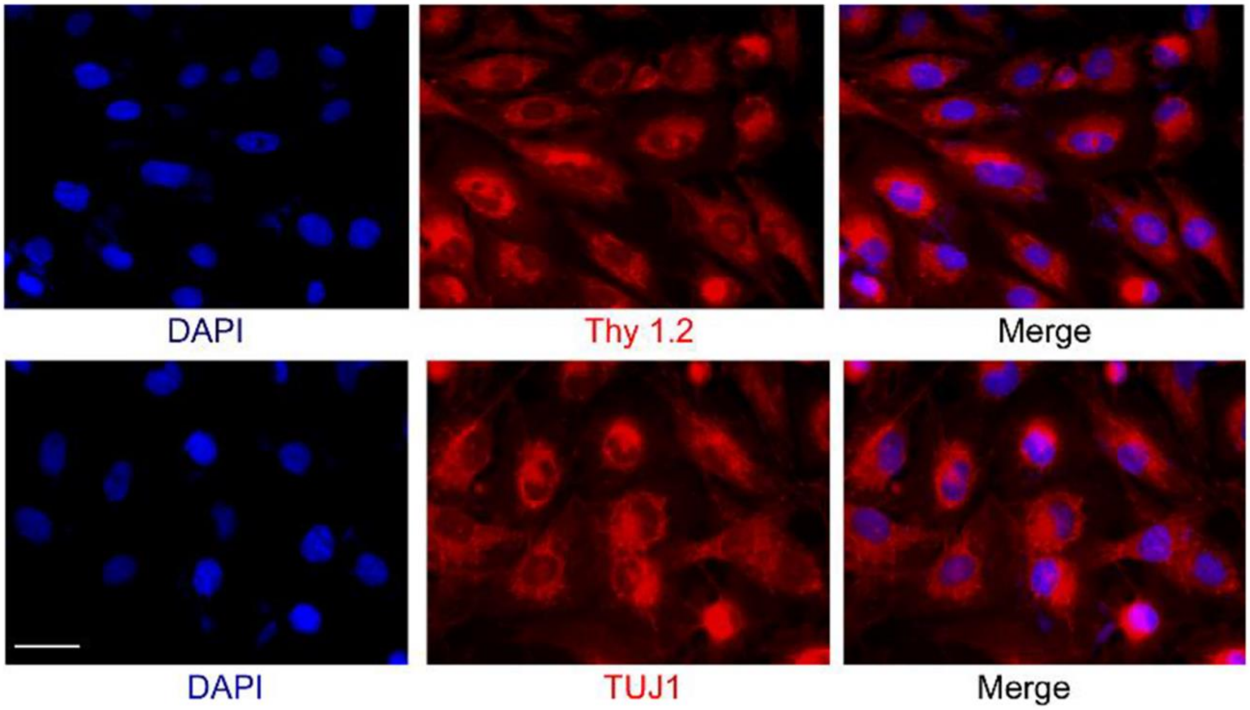

Supplementary Figure 3. Identification of RGCs by immunofluorescence staining with Thy 1.2 and TUJ1. Retinal ganglion cells (RGCs) derived from the newborn mouse retinas by the immunopanning-magnetic separation method. They were then stained with Thy 1.2 and TUJ1 to label RGCs. Nuclei were counterstained with 4',6-diamidino-2-phenylindole (DPAI). Scale bar: 20 μm.

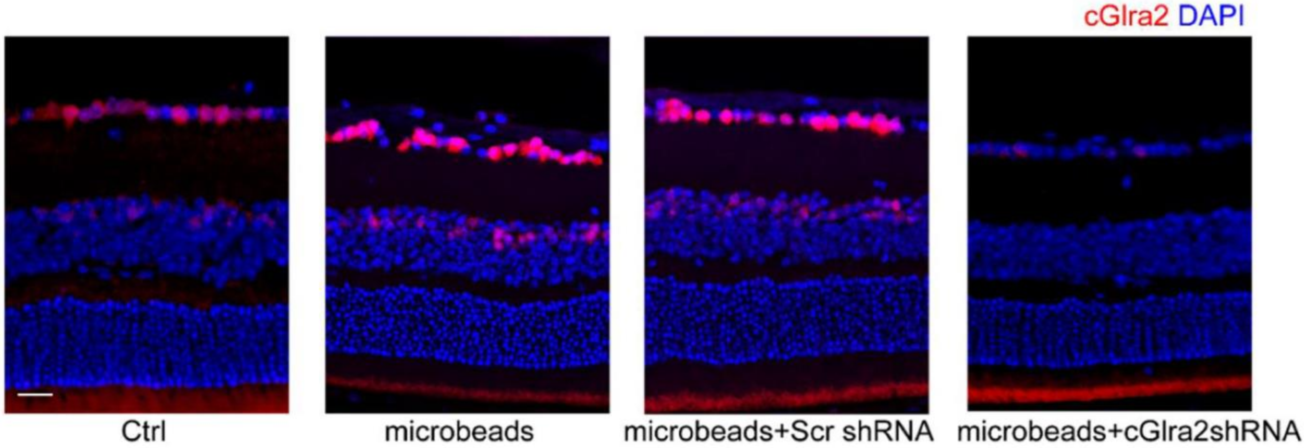

Supplementary Figure 4. Detection of cGlra2 expression distribution in different groups. C57BL/6 mice received intravitreous injections of cGlra2 shRNA, scrambled (Scr) shRNA, or left untreated (Ctrl) for 14 days. The expression distribution of cGlra2 in retinal tissues was detected by FISH assays. Red fluorescence represents the expression pattern of cGlra2 and blue fluorescence is DAPI. Scale bar: Scale bar, 50 μm.

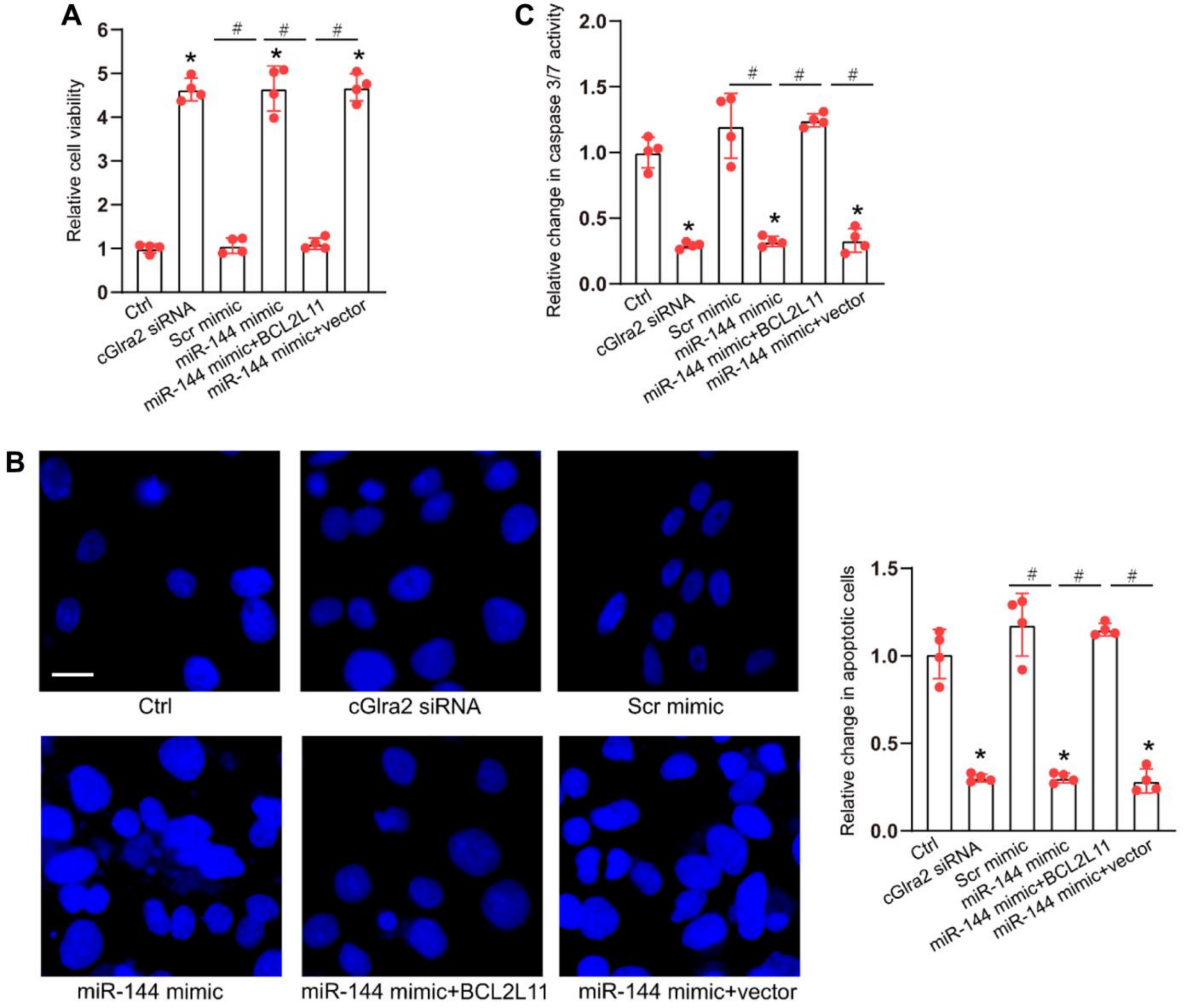

Supplementary Figure 5. cGlra2/miR-144/BCL2L11 signaling axis is involved in regulating RGC function following hydrostatic pressure. (**A**–**C**) RGCs were transfected with Scr siRNA, cGlra2 siRNA, scramble (Scr) mimic, miR-144 mimic, miR-144 mimic plus pcDNA3.1-BCL2L11, miR-144 mimic plus pcDNA3.1 (vector) or left untreated (Ctrl) for 12 h and then exposed to hydrostatic pressure for additional 36 h. RGCs were maintained under the elevated hydrostatic pressure (70 mm Hg) to induce hydrostatic stress. CCK-8 assays were performed to detect RGC viability (**A**, *n* = 4 independent experiments). Hoechst staining and quantification analysis were performed to detect the changes of nuclei morphological characteristics of RGCs (**B**, *n* = 4 independent experiments, Scale bar: 50 μm). Caspase 3/7 activity was performed to detect the degree of RGC apoptosis (**C**, *n* = 4 independent experiments). $^{*}P < 0.05$ vs. Ctrl group; $^{\#}P < 0.05$ between the marked group. All significance was examined using One-way ANOVA followed by Bonferroni's post hoc test.
