# Supplementary material for: Targeting circular RNA-Glra2 alleviates retinal neurodegeneration induced by ocular hypertension
Source: Aging (Albany NY). 2023 Oct 10;15(19):10705–31. doi: 10.18632/aging.205108 (PMC10599745; doi:10.18632/aging.205108)
Supplement: Supplementary Table 1 [file aging-15-205108-s002.doc]

**Supplementary Table 1. Differentially expressed circRNAs between microbeads-injected retinas and saline-injected retinas.**

| ID | logFC | logCPM | LR | PValue | FDR | hostgene |
| --- | --- | --- | --- | --- | --- | --- |
| mmu_circ_0016226 | 4.408706 | 4.686056 | 22.57703 | 2.02E-06 | 0.015762 | Glra2 |
| mmu_circ_0016224 | 4.130122 | 4.696123 | 21.50113 | 3.54E-06 | 0.022086 | Glra2 |
| cicRNA.16113 | 3.810211 | 4.47278 | 15.71962 | 7.35E-05 | 0.211752 | Raph1 |
| mmu_circ_0008388 | 3.668195 | 4.375798 | 13.5516 | 0.000232 | 0.483192 | Dcaf6 |
| cicRNA.3601 | 3.354285 | 4.384591 | 12.35862 | 0.000439 | 0.657974 | Robo2 |
| cicRNA.18946 | 3.280812 | 4.305801 | 10.98304 | 0.000919 | 0.999986 | Snap91 |
| cicRNA.6620 | 3.178197 | 4.555278 | 13.97722 | 0.000185 | 0.407892 | Enox1 |
| mmu_circ_0008386 | 3.151176 | 4.236228 | 9.608711 | 0.001937 | 0.999986 | Dcaf6 |
| mmu_circ_0015587 | 3.093024 | 4.051388 | 7.396572 | 0.006535 | 0.999986 | Grik4 |
| cicRNA.17958 | 3.062999 | 4.086851 | 7.658247 | 0.005651 | 0.999986 | Ptpn4 |
| mmu_circ_0016229 | 3.031877 | 4.998169 | 20.50471 | 5.95E-06 | 0.031324 | Gpm6b |
| cicRNA.10849 | 3.000242 | 4.334913 | 10.15476 | 0.001439 | 0.999986 | Arhgap12 |
| cicRNA.9251 | 2.998391 | 4.511937 | 12.39277 | 0.000431 | 0.657974 | Nln |
| mmu_circ_0011329 | 2.990278 | 4.54624 | 12.81663 | 0.000344 | 0.596577 | Runx1t1 |
| mmu_circ_0008931 | 2.823996 | 4.026303 | 6.3205 | 0.011935 | 0.999986 | 44624 |
| mmu_circ_0002966 | 2.821946 | 4.21337 | 6.664499 | 0.009835 | 0.999986 | Psme4 |
| cicRNA.27483 | 2.787553 | 4.04152 | 6.339014 | 0.011811 | 0.999986 | Ano3 |
| cicRNA.27432 | 2.780084 | 3.930212 | 5.387962 | 0.020276 | 0.999986 | Ryr3 |
| cicRNA.7584 | 2.7495 | 3.986473 | 4.777299 | 0.028837 | 0.999986 | Nek10 |
| cicRNA.25210 | 2.710853 | 4.000434 | 3.943055 | 0.047065 | 0.999986 | Sec61a2 |
| mmu_circ_0005154 | 2.673641 | 4.088888 | 6.373683 | 0.011582 | 0.999986 | Erc2 |
| cicRNA.23088 | 2.660086 | 4.161214 | 6.959837 | 0.008336 | 0.999986 | Dab1 |
| cicRNA.10463 | 2.657641 | 3.969213 | 5.347165 | 0.020756 | 0.999986 | Ptprn2 |
| cicRNA.17599 | 2.652134 | 4.005008 | 5.611682 | 0.017841 | 0.999986 | Lrp5 |
| cicRNA.10465 | 2.648916 | 4.198755 | 7.257932 | 0.007059 | 0.999986 | Ptprn2 |
| cicRNA.16215 | 2.638926 | 3.978953 | 3.94108 | 0.047121 | 0.999986 | Rftn2 |
| mmu_circ_0002003 | 2.618669 | 4.142211 | 5.521773 | 0.018781 | 0.999986 | Grip1 |
| cicRNA.5070 | 2.612077 | 3.918093 | 4.835056 | 0.027887 | 0.999986 | Efcab6 |
| mmu_circ_0012181 | 2.599266 | 4.729153 | 12.82478 | 0.000342 | 0.596577 | Clip2 |
| mmu_circ_0003918 | 2.588307 | 3.914055 | 4.741367 | 0.029446 | 0.999986 | Hdac9 |
| mmu_circ_0007872 | 2.512857 | 3.864833 | 4.205802 | 0.040286 | 0.999986 | Sorcs3 |
| cicRNA.13683 | 2.497744 | 3.887072 | 4.314154 | 0.037797 | 0.999986 | Anks1b |
| mmu_circ_0008921 | 2.496083 | 3.83941 | 3.999403 | 0.045516 | 0.999986 | Bard1 |
| cicRNA.15205 | 2.485946 | 4.028874 | 5.276963 | 0.021609 | 0.999986 | Ptprr |
| cicRNA.20942 | 2.474388 | 3.892651 | 4.289947 | 0.038338 | 0.999986 | Dera |
| cicRNA.10447 | 2.46718 | 3.959818 | 4.724868 | 0.029729 | 0.999986 | Rapgef5 |
| cicRNA.19607 | 2.452746 | 3.87105 | 4.093487 | 0.043049 | 0.999986 | Zcwpw2 |
| mmu_circ_0005231 | 2.452528 | 4.397925 | 8.249312 | 0.004077 | 0.999986 | Sh2d4b |
| cicRNA.7136 | 2.438037 | 3.923411 | 4.395754 | 0.036029 | 0.999986 | Nrg3 |
| mmu_circ_0003879 | 2.430448 | 3.976257 | 4.731213 | 0.02962 | 0.999986 | Myt1l |
| cicRNA.16025 | 2.421362 | 3.992376 | 4.81544 | 0.028206 | 0.999986 | Unc80 |
| cicRNA.6597 | 2.413508 | 3.896598 | 4.155106 | 0.041509 | 0.999986 | Enox1 |
| cicRNA.8478 | 2.411326 | 3.848793 | 3.85022 | 0.049739 | 0.999986 | Shc3 |
| cicRNA.17091 | 2.40424 | 4.005704 | 4.855704 | 0.027555 | 0.999986 | n/a |
| cicRNA.9145 | 2.386522 | 3.860533 | 3.860398 | 0.049439 | 0.999986 | Pde4d |
| mmu_circ_0009728 | 2.378037 | 4.002926 | 4.756223 | 0.029193 | 0.999986 | Ehmt1 |
| cicRNA.2202 | 2.35047 | 3.935089 | 4.229426 | 0.039729 | 0.999986 | Lin54 |
| cicRNA.22161 | 2.338243 | 3.902355 | 3.991826 | 0.045721 | 0.999986 | Snapc3 |
| cicRNA.19329 | 2.315398 | 4.06161 | 4.957562 | 0.025977 | 0.999986 | Slc8a1 |
| cicRNA.24655 | 2.287148 | 3.95823 | 4.195882 | 0.040522 | 0.999986 | Mybpc3 |
| mmu_circ_0000811 | 2.287004 | 4.092525 | 5.073906 | 0.024289 | 0.999986 | Smchd1 |
| mmu_circ_0000197 | 2.28211 | 4.000255 | 4.445888 | 0.034985 | 0.999986 | Anks1b |
| cicRNA.3325 | 2.258294 | 4.070883 | 4.830289 | 0.027964 | 0.999986 | Brwd1 |
| cicRNA.9268 | 2.240541 | 3.922458 | 3.84991 | 0.049749 | 0.999986 | Mast4 |
| cicRNA.11366 | 2.239623 | 4.089721 | 4.892325 | 0.026976 | 0.999986 | Asic2 |
| mmu_circ_0008387 | 2.221993 | 4.789216 | 10.79034 | 0.00102 | 0.999986 | Dcaf6 |
| mmu_circ_0015475 | 2.218084 | 4.475231 | 7.729802 | 0.005432 | 0.999986 | Dpy19l1 |
| mmu_circ_0003676 | 2.19628 | 4.21861 | 5.616329 | 0.017794 | 0.999986 | Unc79 |
| cicRNA.10651 | 2.189083 | 4.002785 | 4.17906 | 0.040926 | 0.999986 | n/a |
| cicRNA.840 | 2.188885 | 4.161083 | 5.189296 | 0.022726 | 0.999986 | Ddx46 |
| mmu_circ_0010070 | 2.178604 | 4.010184 | 4.191618 | 0.040624 | 0.999986 | Kcnh7 |
| cicRNA.27102 | 2.178418 | 4.111104 | 4.820622 | 0.028121 | 0.999986 | Sema6d |
| cicRNA.2201 | 2.133396 | 4.008736 | 4.04503 | 0.044302 | 0.999986 | Wdfy3 |
| mmu_circ_0000407 | 2.126958 | 4.146737 | 4.865756 | 0.027395 | 0.999986 | Kcnk10 |
| cicRNA.15316 | 2.112222 | 4.019735 | 4.043829 | 0.044333 | 0.999986 | Grm1 |
| cicRNA.16710 | 2.054694 | 4.092731 | 4.281224 | 0.038536 | 0.999986 | Syt14 |
| cicRNA.27537 | 1.993134 | 4.162142 | 4.469069 | 0.034514 | 0.999986 | D430041D05Rik |
| mmu_circ_0001934 | 1.973114 | 4.13562 | 4.244592 | 0.039376 | 0.999986 | Syt1 |
| mmu_circ_0003678 | 1.963742 | 4.115252 | 4.098274 | 0.042927 | 0.999986 | Unc79 |
| mmu_circ_0008390 | 1.949899 | 4.388157 | 5.693454 | 0.017028 | 0.999986 | Mpzl1 |
| mmu_circ_0006981 | 1.892937 | 4.258503 | 4.639278 | 0.031248 | 0.999986 | Lbh |
| cicRNA.13659 | 1.872717 | 4.16541 | 4.047502 | 0.044237 | 0.999986 | Sil1 |
| mmu_circ_0008394 | 1.862856 | 4.32263 | 4.884406 | 0.0271 | 0.999986 | Pou2f1 |
| cicRNA.21012 | 1.854309 | 4.251379 | 4.442376 | 0.035057 | 0.999986 | Iqsec3 |
| mmu_circ_0011934 | 1.85268 | 4.762683 | 7.837153 | 0.005118 | 0.999986 | Mapk10 |
| cicRNA.19219 | 1.852342 | 4.29505 | 4.679544 | 0.030524 | 0.999986 | Fgfr1op |
| cicRNA.8506 | 1.79617 | 4.204303 | 3.966752 | 0.046407 | 0.999986 | Fgd3 |
| cicRNA.9087 | 1.795263 | 4.21526 | 4.019144 | 0.044987 | 0.999986 | Ryr2 |
| cicRNA.20664 | 1.781552 | 4.365891 | 4.769267 | 0.028972 | 0.999986 | Prdm5 |
| cicRNA.9237 | 1.769079 | 4.674975 | 6.627639 | 0.010041 | 0.999986 | Adamts6 |
| mmu_circ_0010022 | 1.741883 | 4.234112 | 3.909539 | 0.048013 | 0.999986 | Gpd2 |
| cicRNA.13667 | 1.731441 | 4.354982 | 4.48447 | 0.034204 | 0.999986 | Anks1b |
| cicRNA.9386 | 1.675684 | 4.523234 | 5.135331 | 0.023444 | 0.999986 | Nrxn3 |
| cicRNA.19383 | 1.66448 | 4.437006 | 4.316494 | 0.037745 | 0.999986 | Grik4 |
| cicRNA.8898 | 1.663316 | 4.502296 | 4.953964 | 0.026031 | 0.999986 | Hecw1 |
| cicRNA.9025 | 1.589998 | 4.50383 | 4.587398 | 0.032208 | 0.999986 | Ryr2 |
| cicRNA.2572 | 1.572869 | 4.442565 | 4.201432 | 0.04039 | 0.999986 | Sptan1 |
| mmu_circ_0016443 | 1.499857 | 5.012375 | 6.942194 | 0.008419 | 0.999986 | Uty |
| mmu_circ_0016246 | 1.496268 | 5.33337 | 9.256462 | 0.002347 | 0.999986 | Slc9a7 |
| cicRNA.9385 | 1.487081 | 4.507457 | 4.091892 | 0.043089 | 0.999986 | Nrxn3 |
| mmu_circ_0016223 | 1.4782 | 4.998174 | 6.674788 | 0.009779 | 0.999986 | Ap1s2 |
| cicRNA.26607 | 1.449312 | 4.806103 | 5.347028 | 0.020758 | 0.999986 | Angpt4 |
| mmu_circ_0016250 | 1.428387 | 5.432535 | 9.290035 | 0.002304 | 0.999986 | Slc9a7 |
| mmu_circ_0016241 | 1.402755 | 5.470943 | 9.295175 | 0.002298 | 0.999986 | Slc9a7 |
| mmu_circ_0016248 | 1.38855 | 5.500195 | 9.357824 | 0.00222 | 0.999986 | Slc9a7 |
| mmu_circ_0016242 | 1.348776 | 5.406572 | 8.190282 | 0.004212 | 0.999986 | Slc9a7 |
| mmu_circ_0008389 | 1.332304 | 4.656347 | 3.940398 | 0.04714 | 0.999986 | Dcaf6 |
| mmu_circ_0016243 | 1.239984 | 5.525079 | 7.775589 | 0.005296 | 0.999986 | Slc9a7 |
| mmu_circ_0016244 | 1.194942 | 5.341078 | 6.193057 | 0.012825 | 0.999986 | Slc9a7 |
| mmu_circ_0016236 | 1.169589 | 5.634535 | 7.653811 | 0.005665 | 0.999986 | Mid1 |
| mmu_circ_0016232 | 1.103723 | 5.628331 | 6.832859 | 0.00895 | 0.999986 | Ofd1 |
| cicRNA.26339 | -1.02826 | 5.304951 | 4.417051 | 0.035581 | 0.999986 | Tjap1 |
| mmu_circ_0016424 | -1.02997 | 5.253038 | 4.230837 | 0.039696 | 0.999986 | Rpgr |
| mmu_circ_0008536 | -1.03645 | 5.336786 | 4.610763 | 0.031772 | 0.999986 | Rab3gap2 |
| cicRNA.10040 | -1.04158 | 5.153217 | 3.947693 | 0.046936 | 0.999986 | Nrcam |
| mmu_circ_0008550 | -1.05998 | 5.212655 | 4.304735 | 0.038006 | 0.999986 | Prox1 |
| mmu_circ_0011136 | -1.08244 | 5.380547 | 5.194399 | 0.02266 | 0.999986 | Macf1 |
| mmu_circ_0010713 | -1.11098 | 5.21678 | 4.714642 | 0.029907 | 0.999986 | Nbea |
| cicRNA.20824 | -1.11396 | 5.106343 | 4.284319 | 0.038465 | 0.999986 | Strip2 |
| mmu_circ_0016433 | -1.12832 | 5.498014 | 6.208465 | 0.012714 | 0.999986 | Hdac8 |
| cicRNA.16285 | -1.1341 | 5.200933 | 4.827957 | 0.028002 | 0.999986 | Gls |
| cicRNA.15113 | -1.15783 | 5.383123 | 5.89577 | 0.015177 | 0.999986 | Gns |
| mmu_circ_0016438 | -1.20403 | 5.100772 | 4.917411 | 0.026587 | 0.999986 | Uty |
| mmu_circ_0006194 | -1.23369 | 4.854232 | 4.06468 | 0.043789 | 0.999986 | Pcyt1a |
| mmu_circ_0016222 | -1.23979 | 5.240895 | 5.891818 | 0.015211 | 0.999986 | Ctps2 |
| cicRNA.13913 | -1.24811 | 4.962366 | 4.610392 | 0.031779 | 0.999986 | Sbno2 |
| mmu_circ_0013038 | -1.25214 | 4.859599 | 4.197957 | 0.040473 | 0.999986 | Mical3 |
| mmu_circ_0008541 | -1.25453 | 4.892301 | 4.349477 | 0.03702 | 0.999986 | Gpatch2 |
| mmu_circ_0008443 | -1.26618 | 5.43418 | 7.25574 | 0.007067 | 0.999986 | Fmn2 |
| cicRNA.27296 | -1.28209 | 4.917186 | 4.634664 | 0.031332 | 0.999986 | Ino80 |
| mmu_circ_0008549 | -1.29932 | 4.889267 | 4.620832 | 0.031586 | 0.999986 | Kcnk2 |
| cicRNA.9895 | -1.31526 | 5.18129 | 6.213088 | 0.012681 | 0.999986 | n/a |
| mmu_circ_0005433 | -1.32371 | 4.85955 | 4.64119 | 0.031213 | 0.999986 | Lrch1 |
| mmu_circ_0008555 | -1.34969 | 5.15527 | 6.355543 | 0.011702 | 0.999986 | Rps6kc1 |
| mmu_circ_0004441 | -1.3655 | 4.983412 | 5.530285 | 0.01869 | 0.999986 | Zswim6 |
| mmu_circ_0008552 | -1.36695 | 4.750945 | 4.411009 | 0.035708 | 0.999986 | Tcfap2b |
| mmu_circ_0016439 | -1.42164 | 5.105321 | 6.658442 | 0.009869 | 0.999986 | Uty |
| mmu_circ_0004208 | -1.43907 | 4.571171 | 4.004039 | 0.045391 | 0.999986 | Fut8 |
| cicRNA.6935 | -1.44981 | 5.352779 | 8.60874 | 0.003346 | 0.999986 | Zmym2 |
| cicRNA.21265 | -1.46105 | 4.551577 | 4.026054 | 0.044803 | 0.999986 | Ugdh |
| cicRNA.26086 | -1.479 | 4.596882 | 4.319494 | 0.037678 | 0.999986 | Osbpl2 |
| cicRNA.20426 | -1.53838 | 4.479795 | 4.070658 | 0.043634 | 0.999986 | Mgmt |
| cicRNA.8658 | -1.57182 | 4.596815 | 4.802274 | 0.028422 | 0.999986 | F13a1 |
| mmu_circ_0007944 | -1.57439 | 5.862839 | 15.26406 | 9.35E-05 | 0.250215 | Malat1 |
| cicRNA.16220 | -1.67411 | 4.426686 | 4.433103 | 0.035248 | 0.999986 | Rftn2 |
| cicRNA.14839 | -1.68701 | 5.44326 | 12.09435 | 0.000506 | 0.728939 | Map3k2 |
| cicRNA.21168 | -1.70874 | 4.298004 | 3.943619 | 0.047049 | 0.999986 | Tecrl |
| cicRNA.2626 | -1.72996 | 4.345984 | 4.265612 | 0.038891 | 0.999986 | Prex1 |
| mmu_circ_0015675 | -1.73375 | 4.294024 | 4.022014 | 0.04491 | 0.999986 | Dmxl2 |
| cicRNA.15347 | -1.74176 | 4.373494 | 4.456942 | 0.03476 | 0.999986 | Mettl25 |
| cicRNA.21803 | -1.7419 | 4.420779 | 4.708661 | 0.030011 | 0.999986 | Naa25 |
| mmu_circ_0005789 | -1.75913 | 4.311689 | 4.210681 | 0.04017 | 0.999986 | Top1mt |
| cicRNA.20012 | -1.79411 | 5.053455 | 9.458268 | 0.002102 | 0.999986 | Sorbs2 |
| cicRNA.15390 | -1.82002 | 4.505356 | 5.57185 | 0.018251 | 0.999986 | 2610203C22Rik |
| cicRNA.1733 | -1.90216 | 5.244649 | 12.39169 | 0.000431 | 0.657974 | Astn1 |
| cicRNA.21294 | -1.9056 | 4.239062 | 4.100715 | 0.042865 | 0.999986 | Cdk14 |
| cicRNA.5127 | -2.08498 | 4.979221 | 11.23346 | 0.000803 | 0.999986 | Serhl |
| mmu_circ_0008531 | -2.09813 | 4.046581 | 3.98015 | 0.046039 | 0.999986 | Mosc1 |
| cicRNA.8712 | -2.19637 | 4.142006 | 4.865205 | 0.027404 | 0.999986 | Gmds |
| cicRNA.17441 | -2.30678 | 4.037137 | 4.55008 | 0.032917 | 0.999986 | Cacna1e |
| mmu_circ_0002815 | -2.3362 | 5.090031 | 14.80618 | 0.000119 | 0.297654 | Gaa |
| cicRNA.3661 | -2.34254 | 4.66415 | 9.815451 | 0.001731 | 0.999986 | Cggbp1 |
| cicRNA.21644 | -2.39507 | 3.884143 | 3.847075 | 0.049833 | 0.999986 | Cux1 |
| cicRNA.1613 | -2.40736 | 4.113813 | 5.384991 | 0.020311 | 0.999986 | Cyp20a1 |
| cicRNA.26505 | -2.42604 | 4.26365 | 6.59293 | 0.010238 | 0.999986 | Uqcc1 |
| cicRNA.16526 | -2.55866 | 4.040458 | 5.328685 | 0.020977 | 0.999986 | Dst |
| cicRNA.14344 | -2.56025 | 4.321877 | 7.64257 | 0.005701 | 0.999986 | Mcu |
| cicRNA.19866 | -2.59296 | 4.034015 | 5.211517 | 0.022438 | 0.999986 | Slc10a7 |
| cicRNA.27059 | -2.62727 | 4.863834 | 14.12916 | 0.000171 | 0.399749 | Runx2 |
| cicRNA.2918 | -2.64841 | 4.751338 | 12.78059 | 0.00035 | 0.596577 | Btbd9 |
| cicRNA.26231 | -2.75196 | 4.02298 | 5.764496 | 0.016353 | 0.999986 | Cse1l |
| cicRNA.3397 | -3.10621 | 4.885193 | 17.91986 | 2.30E-05 | 0.086342 | Dopey2 |
| cicRNA.21404 | -3.18823 | 5.09435 | 22.49851 | 2.10E-06 | 0.015762 | Gm29609 |
| cicRNA.27379 | -3.33945 | 4.320292 | 10.76608 | 0.001034 | 0.999986 | Gm26917 |
| cicRNA.9868 | -3.38976 | 4.476992 | 13.07413 | 0.000299 | 0.590516 | Fam179b |
| cicRNA.12320 | -3.81932 | 4.581201 | 16.61483 | 4.58E-05 | 0.142999 | Kremen1 |
| cicRNA.17058 | -4.16 | 4.541614 | 17.31132 | 3.17E-05 | 0.108093 | Pld5 |
| cicRNA.1949 | -4.163 | 4.80378 | 22.56619 | 2.03E-06 | 0.015762 | Bbs9 |
| cicRNA.7215 | -4.26402 | 4.67409 | 20.28075 | 6.69E-06 | 0.031324 | Ankrd28 |
| cicRNA.27992 | -4.37135 | 4.601007 | 19.24611 | 1.15E-05 | 0.047844 | 9130011E15Rik |
| mmu_circ_0016436 | -4.49839 | 4.886129 | 26.08976 | 3.26E-07 | 0.006107 | Uty |
