# Supplementary material for: Targeting circular RNA-Glra2 alleviates retinal neurodegeneration induced by ocular hypertension
Source: Aging (Albany NY). 2023 Oct 10;15(19):10705–31. doi: 10.18632/aging.205108 (PMC10599745; doi:10.18632/aging.205108)
Supplement: Supplementary Table 2 [file aging-15-205108-s003.pdf]

SUPPLEMENTARY TABLE

Supplementary Table 2. Baseline demographics and characteristics of involved patients.

	Glaucoma	Cataract
Age	59.3 ± 7.5	62.8 ± 6.9
Gender (Male/Female)	12/8	11/9
IOP, mm Hg	24.2 ± 3.9	10.5 ± 1.7
Ocular comorbidity	None	None
Eye laterality (Right/Left)	13/7	8/12
